# Identification of genetic markers of quinine partial resistance in *Plasmodium falciparum*

**DOI:** 10.1038/s41564-026-02410-7

**Published:** 2026-07-06

**Authors:** Mariko Kanai, Sachel Mok, Tomas Yeo, Melanie J. Shears, Jin H. Jeon, Sunil K. Narwal, Leila S. Ross, Kharizta Wiradiputri, Meseret T. Haile, Abhai K. Tripathi, Godfree Mlambo, Jonathan Kim, Eva Gil-Iturbe, Heekuk Park, Tolla Ndiaye, John Okombo, Kurt E. Ward, Felix D. Rozenberg, Kate J. Fairhurst, Sydney M. Gavula, Talia S. Bloxham, Jessica L. Bridgford, Tanaya Sheth, Manuel Llinás, Marcus C. S. Lee, Jennifer L. Small-Saunders, Filippo Mancia, Matthias Quick, Anne-Catrin Uhlemann, Photini Sinnis, David Armand Fidock

**Affiliations:** 1 Department of Microbiology and Immunology, Columbia University Irving Medical Center, New York, NY USA; 2 Center for Malaria Therapeutics and Antimicrobial Resistance, Columbia University Irving Medical Center, New York, NY USA; 3 Division of Infectious Diseases, Department of Medicine, Columbia University Irving Medical Center, New York, NY USA; 4 Department of Molecular Microbiology and Immunology, Johns Hopkins Bloomberg School of Public Health, Baltimore, MD USA; 5 Johns Hopkins Malaria Research Institute, Johns Hopkins University, Baltimore, MD USA; 6 Department of Physiology and Cellular Biophysics, Columbia University Irving Medical Center, New York, NY USA; 7 Department of Psychiatry, Columbia University Irving Medical Center, New York, NY USA; 8 Department of Chemistry, The Pennsylvania State University State College, University Park, PA USA; 9 Huck Center for Malaria Research, The Pennsylvania State University State College, University Park, PA USA; 10 Department of Biochemistry and Molecular Biology, The Pennsylvania State University State College, University Park, PA USA; 11 Wellcome Sanger Institute, Wellcome Genome Campus, Hinxton, UK; 12 Biological Chemistry and Drug Discovery, University of Dundee, Dundee, UK; 13 Area Neuroscience - Molecular Therapeutics, New York State Psychiatric Institute, New York, NY USA; 14 Department of Parasites and Insect Vectors, Institut Pasteur, Paris, France

**Keywords:** Parasite genetics, Parasite genomics

## Abstract

The genetic basis of *Plasmodium falciparum* resistance to quinine, a drug used to treat severe malaria, has long been unclear. To investigate this, here we used a human liver-chimaeric mouse model to conduct a *P. falciparum* genetic cross between quinine-partially resistant and quinine-sensitive parasites. Drug profiling and quantitative trait loci analyses of 120 unique recombinant progeny mapped resistance to segments on chromosomes 7 and 12, indicating a polygenic basis. The chloroquine resistance transporter PfCRT and a structurally similar putative drug/metabolite transporter, DMT1, were identified as primary chromosome 7 candidates based on gene-editing studies. In a proteoliposome assay, both mutant DMT1 and PfCRT transported more quinine than their wild-type isoforms. DMT1 localized to the *P. falciparum* digestive vacuole, lipid bodies, parasitophorous vacuolar membrane and structures associated with vesicular trafficking. An ATP-dependent zinc metalloprotease (FtsH1) on chromosome 12 also modulated quinine and chloroquine resistance. We suggest that genotypic surveillance of these markers should be performed in clinical settings of quinine use.

## Abstract (web summary)

A genetic cross of *Plasmodium falciparum* parasites revealed that quinine resistance is polygenic and is associated with mutations in transporter proteins on chromosome 7 (PfCRT and DMT1) and a metalloprotease on chromosome 12 (FtsH1), highlighting key markers for clinical surveillance.

## Main

In 2024, malaria caused an estimated 282 million cases and an estimated 610,000 deaths^1^. Treatment of *Plasmodium falciparum* uncomplicated malaria depends on artemisinin-based combination therapies (ACTs), which combine fast-acting yet short-lived artemisinin derivatives with longer-lasting partner drugs. Artesunate is the first-line treatment for severe malaria.

Alarmingly, artemisinin partial resistance, which arose in Southeast Asia, has now emerged in multiple African countries^2,3^. The threat of widespread artemisinin resistance in Africa and loss of ACT efficacy underscores the urgent need for alternative drugs with distinct modes of action. Trials are underway with several candidates^4^; however, deployment remains years away. A potential alternative is to reintroduce licensed drugs such as quinine, including for the treatment of severe malaria. Quinine is also recommended for treating *P. falciparum* infections in first-trimester pregnant women^5,6^. This drug has largely retained efficacy since its introduction in the 1600s. Nonetheless, low-grade quinine resistance was first reported in ~1910 in Brazil, with subsequent reports from South America, Africa and Southeast Asia^7–10^. Quinine treatment failure may result from parasite partial resistance or other factors including variability in pharmacokinetics, compliance and drug quality^11,12^.

**Extended Data Fig. 1:**
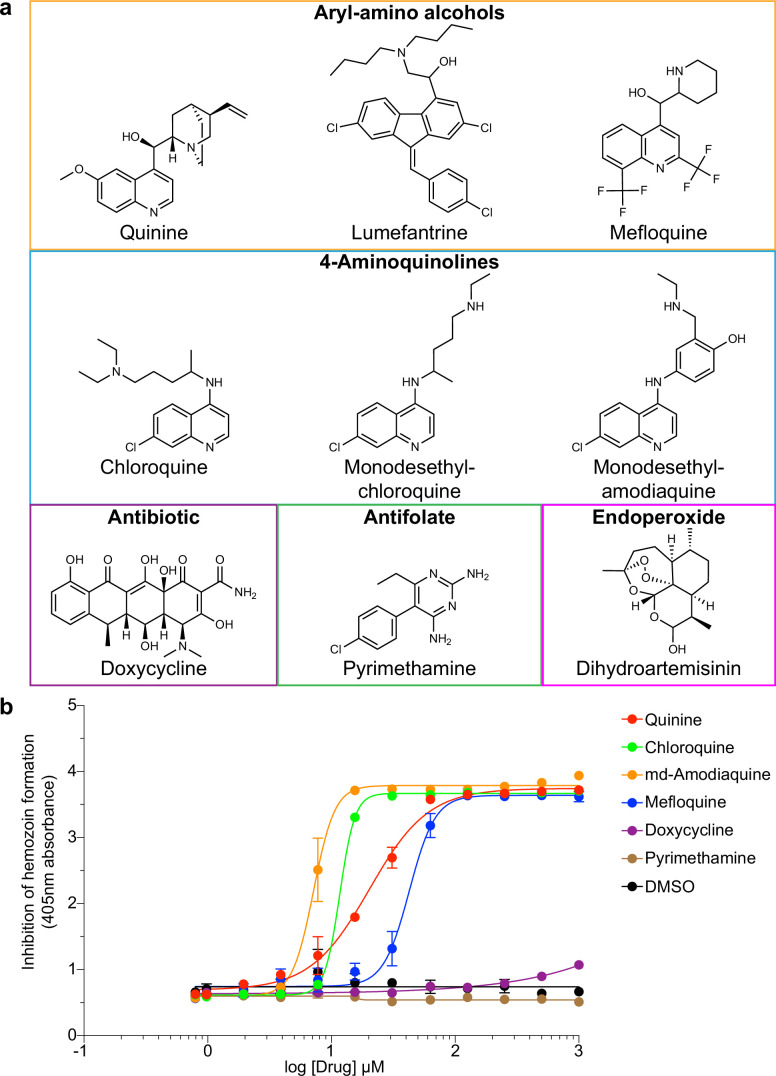
Quinine inhibits β-haematin formation to a lesser degree than chloroquine but more than mefloquine in a cell-free system. **a**, Chemical structures of the antiplasmodial compounds used in this study. Quinine, lumefantrine, and mefloquine are aryl-amino alcohols; chloroquine, monodesethyl-chloroquine, and monodesethyl-amodiaquine are 4-aminoquinolines; doxycycline is a tetracycline-class antibiotic; pyrimethamine is an antifolate; dihydroartemisinin is an active metabolite of artemisinin, a sesquiterpene lactone endoperoxide. Quinine and mefloquine have the aminoquinoline ring found in chloroquine, monodesethyl-chloroquine, and monodesethyl-amodiaquine. Chemical formulas, SMILES, and Tanimoto scores of these compounds are indicated in Supplementary Table 15. The primary sites of action of QN, LMF, and MFQ remain unknown. Chloroquine and amodiaquine concentrate into the parasite digestive vacuole, wherein they bind toxic haem and prevent their detoxification into inert haemozoin. Artemisinins are activated by Fe^2+^ haem produced during haemoglobin degradation in the DV. **b**, Representative concentration-dependent inhibition of β-haematin formation by quinine, chloroquine, md-amodiaquine, mefloquine, two non-haem detoxification pathway-acting negative control antimalarials (doxycycline and pyrimethamine), and DMSO. Mean ± s.e.m. IC_50_ values from *N* = 3 experiments are listed in Supplementary Table 14. Source data

The genetic basis of quinine partial resistance remains incompletely understood. Limited associations have been reported with the *P. falciparum* chloroquine resistance transporter gene (*pfcrt*, via single nucleotide polymorphisms (SNPs)) and multidrug resistance protein 1 gene (*pfmdr1*, via SNPs or copy-number variations (CNVs)). Both *pfcrt* and *pfmdr1* encode membrane transporters on the parasite’s digestive vacuole (DV), an acidic compartment where haemoglobin is proteolytically degraded and multiple antimalarials inhibit the detoxification of haem. Variants in *pfcrt* and *pfmdr1* are key determinants of parasite susceptibility to antimalarials including chloroquine, amodiaquine and lumefantrine. The sodium/hydrogen exchanger gene (*pfnhe-1*) and the HECT E3 ubiquitin ligase *pfut* have also been potentially implicated in quinine partial resistance^13–18^. Quinine is an aryl-amino alcohol, related to the ACT partner drugs mefloquine and lumefantrine (Extended Data Fig. 1a), which share partially overlapping yet still enigmatic modes of action^19^.

Genetic crosses are a proven approach to identify drug resistance determinants in *P. falciparum*^20–22^. This methodology has been empowered by the development of a human liver-chimaeric mouse model^23^. We applied this model to explore *P. falciparum* resistance to quinine, as well as the former first-line 4-aminoquinoline drug chloroquine (Extended Data Fig. 1a).

## Results

### Implementing a genetic cross to map markers of quinine and chloroquine resistance

To identify markers of quinine resistance, we implemented a genetic cross between Cam3.II and NF54 parasites (Extended Data Table 1). Cam3.II, isolated from western Cambodia^24,25^, is chloroquine- and partially quinine-resistant. This parasite expresses genetic variants of *pfcrt* and *pfut* previously implicated in quinine resistance, and harbours two copies of the *pfmdr1* N86/Y184F variant^15,18,26,27^ (Extended Data Table 1). NF54, from east Africa^28^, is sensitive to both drugs and is wild type (WT) at these loci. Mean quinine and chloroquine 50% inhibitory concentration (IC_50_) values were ~90 nM for Cam3.II and ~10 nM for NF54 in media with human serum, and ~118 nM for Cam3.II and ~14 nM for NF54 in media with Albumax (no serum).

**Extended Data Fig. 2:**
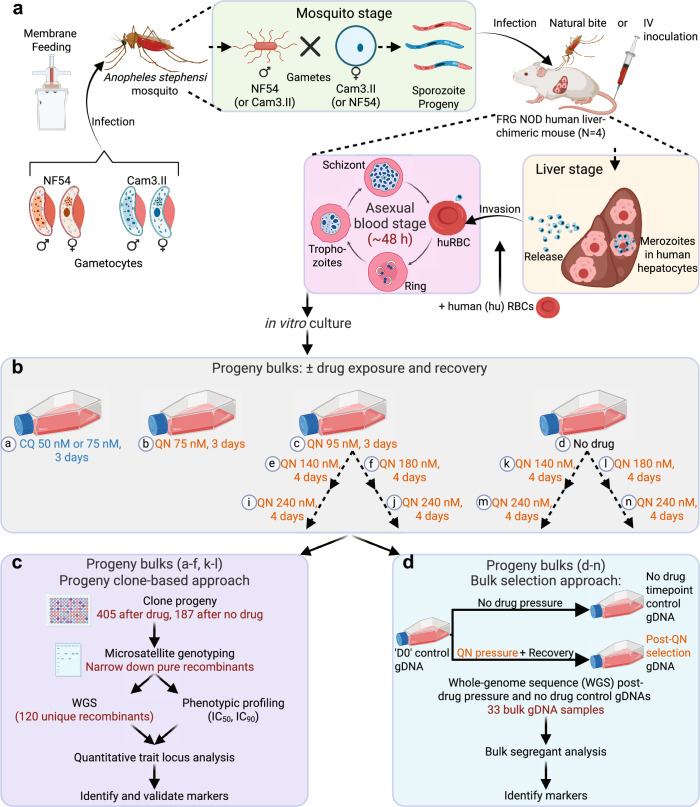
Workflow of the NF54 × Cam3.II genetic cross, isolation of progeny, and bulk selection vs. progeny clone-based trait mapping. **a**, Overview of the genetic cross performed using *Anopheles stephensi* mosquitoes and FRG NOD human-liver chimeric mice (*N* = 4). RBCs, red blood cells. **b**, Drug selection conditions were applied to progeny bulks, followed by a drug-free recovery period after which bulk genomic DNA was harvested and/or the progeny were cloned out. **c**, **d**, Genetic determinants of drug resistance were identified using progeny clone-based linkage approaches (**c**) or bulk selection approaches with bulk segregant analysis (**d**). Images were created with BioRender.com. The NF54 and Cam3.II parents are described in Extended Data Table 1, the mosquito and mouse infections are described in Supplementary Table 1, and the total number of clonal progeny obtained are indicated in Supplementary Table 19. Created in BioRender; Kanai, M. https://BioRender.com/u77n657 (2026).

For this cross, we fed mature NF54 and Cam3.II gametocytes to female *Anopheles stephensi* mosquitoes^29^ (Extended Data Fig. 2a), enabling meiotic recombination and production of haploid salivary gland sporozoites (Supplementary Table 1). We infected four FRG NOD human liver-chimaeric (huHep) mice with ~60,000–200,000 sporozoites each via mosquito bite or intravenous inoculation. After 6 days of liver-stage development, human red blood cells (RBCs) were infused and on day 7, blood was drawn to capture haploid asexual blood-stage (ABS) parasites.

### Combining bulk progeny cloning with differential drug selection yields 120 unique recombinant progeny

We expanded the four mouse progeny bulk cultures in serum-containing media with or without various concentrations of quinine or chloroquine for 3–4 days and recovered clones by limiting dilution (Extended Data Fig. 2b,c). Recombinants were identified by SNP and microsatellite genotyping and confirmed by whole-genome sequencing (WGS).

For each cloned progeny, we classified nucleotide variants at the 13,116 SNP positions that distinguish the parents (Supplementary Table 2). Of these, 1,836 SNPs were used to construct a genetic map, yielding a recombination rate of 13.7 kilobases of physical distance per centimorgan of genetic distance (kb cM^−1^), consistent with previous crosses^22,23,30–33^ (Supplementary Table 3). A total of 55 unique recombinant progeny were obtained without drug pressure. Of these, 45 (82%) carried WT *pfcrt*, consistent with its fitness advantage over mutant *pfcrt* in the absence of chloroquine pressure^34^.

**Fig. 1:**
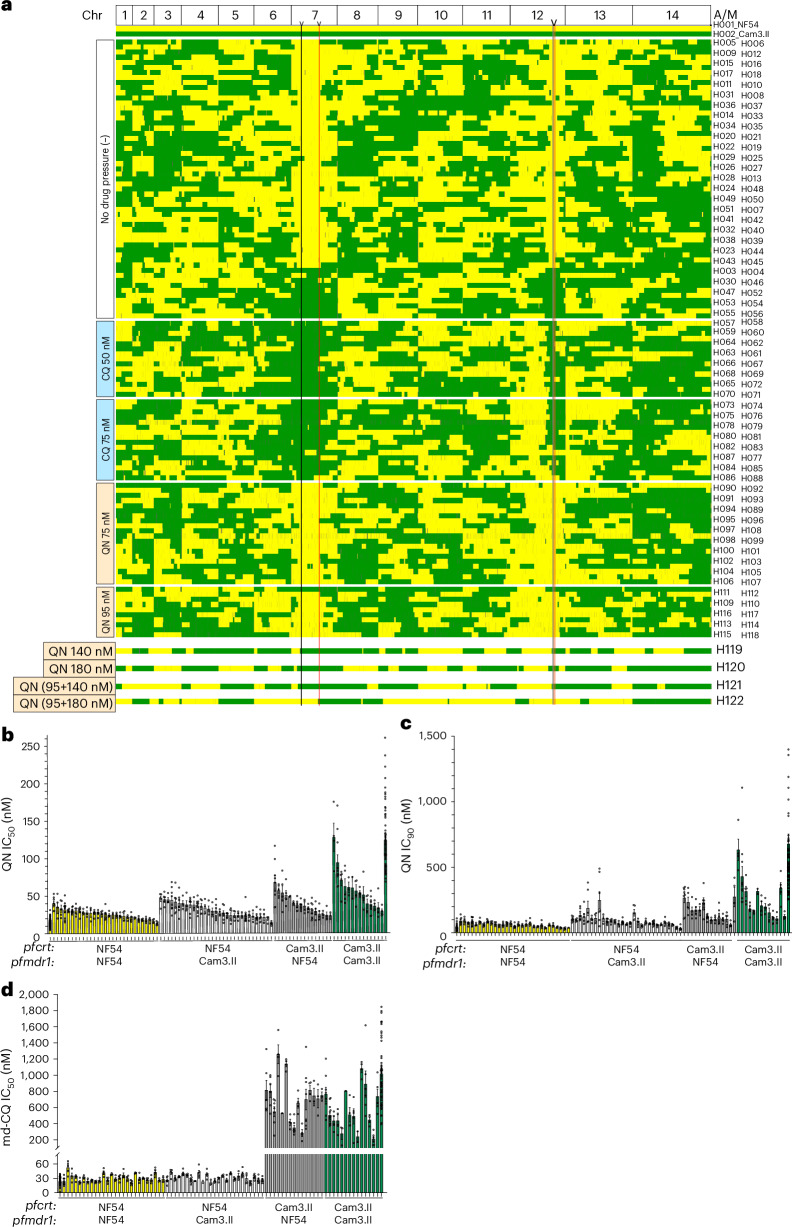
A *P. falciparum* NF54 × Cam3.II genetic cross yielded 120 independent recombinant progeny with diverse genomic and drug resistance profiles. **a**, Recombinant progeny representing each of 120 unique haplotypes were hierarchically clustered by similarity based on their NF54 (yellow) or Cam3.II (green) alleles at 13,116 SNP positions within each selection condition: no drug pressure, chloroquine (CQ) 50 nM or 75 nM, or quinine (QN) 75 nM, 95 nM, 140 nM, 180 nM, (95 nM + 140 nM), (95 nM + 180 nM) or (95 nM + 140 nM + 240 nM), in that order. Some haplotypes were obtained in more than one selection condition (Supplementary Table 4). NF54 (H001) and Cam3.II (H002) parents are shown on top. H, haplotype; A/M, apicoplast/mitochondria (these were always co-inherited); black line, *pfcrt*; red line, *dmt1*; grey line, *ftsh1*; red box, quinine IC_90_, monodesethyl-chloroquine (md-CQ) IC_90_, chloroquine IC_50_ and IC_90_ chromosome 12 QTL locus. **b**–**d**, Mean ± s.e.m. quinine IC_50_ (**b**) and IC_90_ (**c**) values, and md-chloroquine IC_50_ values (**d**). *N* = 1–85 biological replicates, with a majority of >4, always with technical duplicates. IC, s.e.m. and *N* values are listed in Supplementary Table 4 and the Source data. Data were generated with parasites cultured in media with Albumax (no serum). Source data

Quinine or chloroquine pressure (Extended Data Fig. 2b) yielded 65 additional unique recombinant progeny (Fig. 1a). Twenty-six were obtained after quinine selection at ≥95 nM (∼1× Cam3.II IC_50_), of which 17 (65%) had WT *pfcrt* (Supplementary Fig. 1a). Two to three rounds of increasing quinine pressure yielded 4 rare haplotypes. All chloroquine-selected progeny carried mutant Cam3.II (‘Dd2’ variant) *pfcrt* (Supplementary Fig. 1a), which encodes the primary chloroquine resistance determinant^15,27,35–38^. Only 5 haplotypes were recovered under both quinine and chloroquine selections, suggesting non-identical resistance mechanisms.

**Extended Data Fig. 3:**
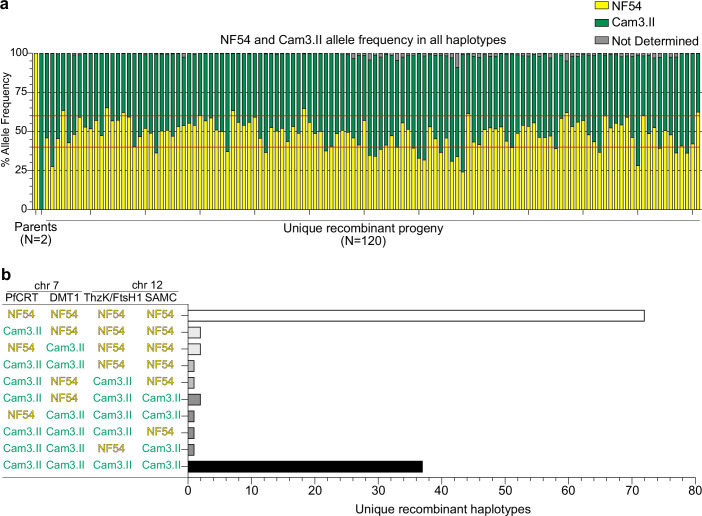
Genome-wide frequencies and *pfcrt*, *dmt1*, *thzk*/*ftsh1*, and *samc* allele representation in the recombinant progeny. **a**, Percentage of NF54 and Cam3.II allele frequencies averaged across the genome in 13,116 SNP positions for each of the parents and 120 recombinant progeny, ordered by haplotype number. **b**, Number of unique recombinant progeny with the wild-type NF54 or mutant Cam3.II alleles for *pfcrt*, *dmt1*, *thzk*/*ftsh1*, and *samc*. The mutant Cam3.II alleles for each gene are listed in Supplementary Table 9. Source data

Across all conditions, we obtained 120 unique recombinant haplotypes, with roughly equal contributions of parental alleles when averaged across their genomes (Fig. 1a, Supplementary Table 4 and Extended Data Fig. 3).

### Bulk segregant analysis identifies two quantitative trait loci peaks on chromosome 7 that associate with quinine resistance

**Extended Data Fig. 4:**
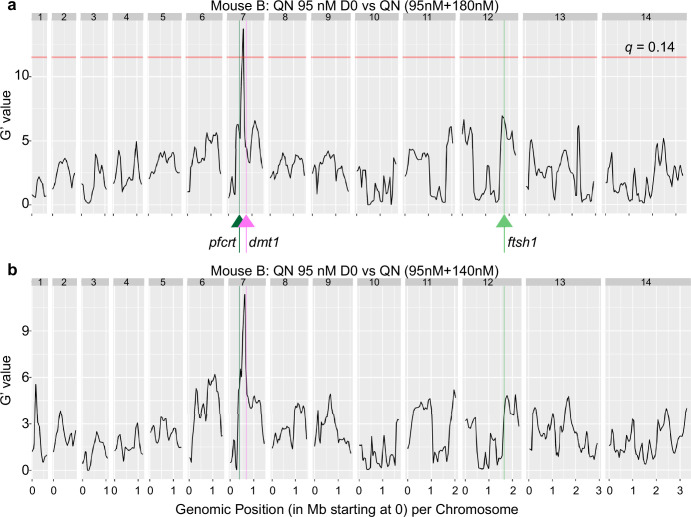
Bulk segregant analyses of progeny pools pressured with quinine or no-drug controls. **a**, **b**, Genetic loci enriched by quinine (QN) in drug-no drug pairwise comparisons. Black lines are G’ values comparing allele frequency between QN-pressured bulk progeny and control non-drug-pressured bulk progeny. The red line indicates the False Discovery Rate at the indicated threshold. Samples are described in Supplementary Table 5. Quantitative trait locus regions are described in Supplementary Table 6. Of note, mutant *pfcrt*/mutant *ftsh1*, or WT *pfcrt/*WT *ftsh1*, continued to be co-inherited in the QN-pressured progeny, as was seen in the non-drug-pressured progeny.

To map chromosomal regions associated with quinine partial resistance, we performed WGS of quinine-pressured parasite pools. We then conducted bulk segregant analysis by comparing genome data of quinine-pressured versus ‘no-drug timepoint control’ or day 0 (D0) controls (Extended Data Fig. 2b,d and Supplementary Table 5). By analysing parasites pressured up to 180 nM quinine, we observed a significant quantitative trait locus (QTL) peak on chromosome 7 (spanning 58 kb), located 191 kb downstream of *pfcrt* and driven by inheritance of the Cam3.II sequence (Extended Data Fig. 4 and Supplementary Table 6). This QTL did not contain any previously known resistance markers. This peak was also observed in parasites pressured up to 140 nM quinine (Extended Data Fig. 4).

### Progeny clone-based trait mapping identifies resistance-associated peaks on chromosomes 7 and 12

**Fig. 2:**
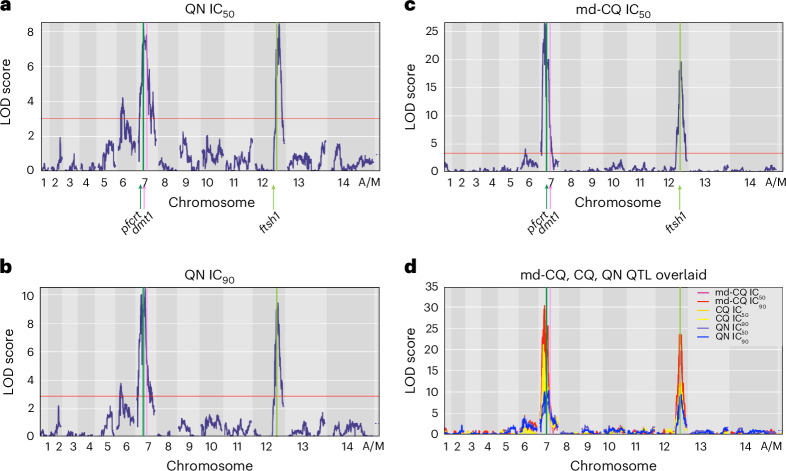
QTL mapping of quinine, chloroquine and md-chloroquine susceptibilities in the progeny and parents reveals a non-*pfcrt*-centred chromosome 7 quinine IC_90_ peak and an additional shared peak on chromosome 12. **a**–**c**, LOD plots for geometric mean quinine IC_50_ (**a**), quinine IC_90_ (**b**) and md-chloroquine IC_50_ (**c**) values using data associated with the cross parents and 90, 85 and 80 progeny, respectively. The red line indicates the 95% probability threshold. **d**, md-chloroquine, chloroquine and quinine LOD plots overlaid. Chromosome 7: *pfcrt* (dark green line), *dmt1* (pink line); chromosome 12: *thzk* and *ftsh1* (light green line).

**Extended Data Fig. 5:**
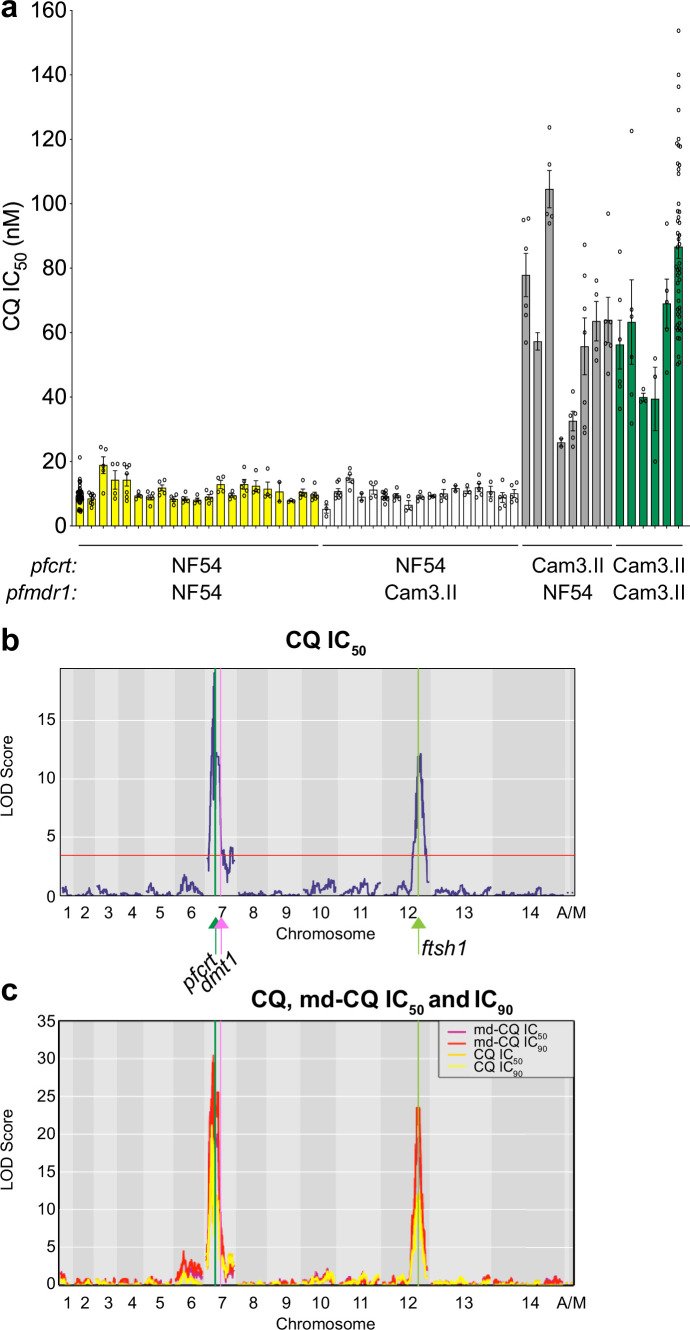
Phenotypic response of parents and recombinant progeny to chloroquine and monodesethyl-chloroquine closely align. **a**, Chloroquine (CQ) response as measured by mean ± s.e.m. IC_50_ values (*N* = 2 − 50 biological replicates, with technical duplicates). IC_50_, SEM, and N values are listed in Supplementary Table 4 and the Source data. **b**, Logarithm of the odds (LOD) plot for geometric mean CQ IC_50_ values of the parents and 50 profiled progeny. The red line indicates the 95% probability threshold. **c**, Overlay of monodesethyl-chloroquine (md-CQ) and CQ LOD plots. A/M, apicoplast/mitochondria (these are always coinherited). The highest LOD peaks for CQ and md-CQ align with *pfcrt* on chromosome 7 and the segment including *ftsh1* on chromosome 12. Note that *dmt1* was not within the dominant chromosome 7 peak for CQ or md-CQ, unlike for quinine. Source data

To independently map chromosomal segments associated with quinine resistance, we measured quinine IC_50_ and IC_90_ values of individually cloned progeny in 72-h assays (Fig. 1b,c and Supplementary Table 4). Phenotypic data were then compared with whole-genome sequences to identify QTLs (Fig. 2a,b,d and Supplementary Table 7). As comparators, we included chloroquine and its metabolite monodesethyl-chloroquine (md-chloroquine) (Figs. 1d and 2c,d, and Extended Data Fig. 5).

For quinine, we obtained the highest, statistically significant QTL peaks on chromosomes 7 and 12, and several other minor QTLs with lower logarithm of the odds (LOD) scores, providing evidence that quinine resistance is multigenic (Fig. 2a,b,d). The quinine IC_50_ chromosome 7 peak was centred 63 kb downstream of *pfcrt*. An overlapping quinine IC_90_ (high-grade resistance) peak was centred 296 kb downstream of *pfcrt* and encompassed 82 genes, of which 33 genes harboured non-synonymous SNPs between the parents (Supplementary Tables 7 and 8). The quinine QTLs on chromosome 12 closely overlaid those separately observed with chloroquine and md-chloroquine. The chromosome 7 segment observed with our cloned progeny-based QTL overlaid that observed with uncloned bulk cultures, whereas the chromosome 12 peak was only identified using our higher-resolution progeny-based analysis.

For md-chloroquine and chloroquine, only two QTLs were identified, both of which were shared with quinine—a chromosome 7 peak harbouring *pfcrt* and a chromosome 12 peak that was usually co-inherited with *pfcrt* (that is mostly mutant or WT at both loci, indicating strong linkage disequilibrium; Fig. 2c,d, Supplementary Tables 4 and 7 and Extended Data Fig. 5). The downstream chromosome 7 peak observed with quinine was not included in the *pfcrt*-centred smaller peak detected for chloroquine and md-chloroquine, arguing for a role for this downstream peak in quinine but not chloroquine resistance.

### *dmt1* is a candidate quinine resistance determinant on chromosome 7

**Extended Data Fig. 6:**
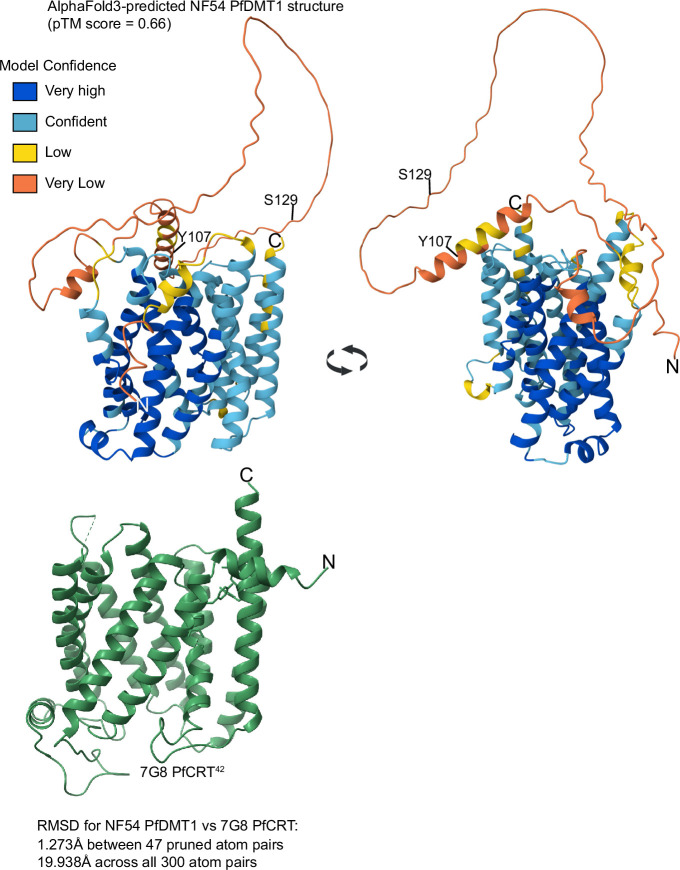
PfDMT1 is structurally similar to another drug metabolite transporter superfamily protein, PfCRT. Comparison of the (top) AlphaFold3-predicted NF54 DMT1 structure^41^ and the (bottom) cryo-EM-derived 7G8 PfCRT structure^42^. The predicted template modeling (pTM) is indicated for the AlphaFold3 NF54 DMT1 structure, which exceeds the 0.5 threshold for predicted fold reliability. The Y107 and S129 positions are indicated on the AlphaFold DMT1 structure. Root mean square deviation (RMSD) scores, calculated using ChimeraX MatchMaker, are indicated for PfCRT and DMT1.

Among the 33 polymorphic genes in the dominant chromosome 7 QTL, we identified *dmt1*, which had a LOD of 10.4 and was only ~4 kb upstream of the peak LOD signal in the QTL segment. DMT1 is annotated as a putative drug/metabolite transporter that, similar to PfCRT, is a member of the drug/metabolite exporter family within the DMT superfamily. DMT1 has 434 amino acids with 9 or 10 predicted transmembrane domains^39,40^, whereas PfCRT has 424 amino acids and 10 transmembrane domains (Extended Data Fig. 6). Cam3.II DMT1 harbours the Y107N and S129L mutations that are absent in NF54. Both parents harbour a single copy of *dmt1*.

To further explore the QTL associations between *pfcrt*, *dmt1* and resistance to quinine or chloroquine, we performed additional QTL analyses by fixing either gene as a covariate (Supplementary Fig. 2). Results with quinine showed a large reduction in the LOD score for both chromosome 7 loci, contrasting with chloroquine where the LOD was substantially reduced for *pfcrt* only, with no impact on *dmt1*. For quinine, results with *pfcrt* as a covariate also identified a chromosome 5 peak that includes *pfmdr1* as a potential minor contributor (Supplementary Fig. 2). This peak was not observed for md-chloroquine. These data agree with earlier gene-editing studies in a chloroquine-resistant (*pfcrt* mutant) parasite with two *pfmdr1* copies (present in Cam3.II but not in NF54) that associated with elevated quinine but not chloroquine IC_50_ values^26^.

**Fig. 3:**
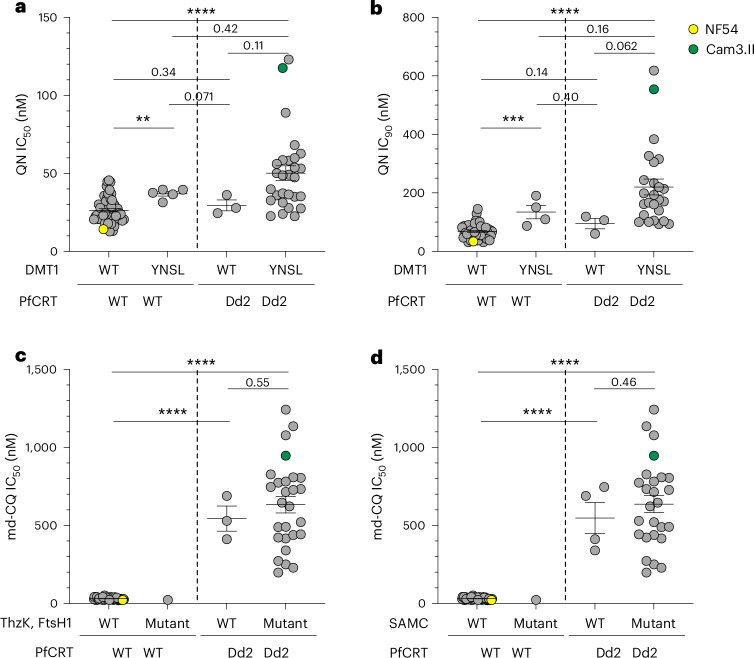
Identification of the top candidate genes from the quinine chromosome 7 and quinine, chloroquine, md-chloroquine chromosome 12 QTL peaks. **a**–**d**, Quinine (**a**,**b**) and md-chloroquine (**c**,**d**) mean ± s.e.m. IC levels for the profiled unedited cross progeny and two parents, grouped by *pfcrt*, *dmt1*, *thzk*, *ftsh1* and *samc* genotypes. Statistical significance was determined using two-sided Mann–Whitney *U*-tests (*N* = 1–27 biological replicates, with technical duplicates). YNSL, Y107N/S129L mutation. IC, s.e.m. and *N* values are listed in the Source data. For **a**, WT PfCRT, mutant DMT1 versus WT PfCRT, WT DMT1, *P* = 0.0023; mutant PfCRT, mutant DMT1 versus WT PfCRT, WT DMT1, *P* < 0.0001. For **b**, WT PfCRT, mutant DMT1 versus WT PfCRT, WT DMT1, *P* = 0.0005; mutant PfCRT, mutant DMT1 versus WT PfCRT, WT DMT1, *P* < 0.0001. For **c**, mutant PfCRT, WT ThzK/FtsH1 versus WT PfCRT, WT ThzK/FtsH1 and mutant PfCRT, mutant ThzK/FtsH1 versus WT PfCRT, WT ThzK/FtsH1, *P* < 0.0001. For **d**, mutant PfCRT, WT SAMC versus WT PfCRT, WT SAMC, and mutant PfCRT, mutant SAMC versus WT PfCRT, WT SAMC, *P* < 0.0001. **P < 0.01, ***P < 0.001, ****P < 0.0001. Source data

When stratifying results based on the *pfcrt* allele, we observed a chromosome 7 QTL peak with quinine IC_90_ analyses for *dmt1* only in the subset of WT but not mutant *pfcrt* parasites. Nonetheless, 25/28 *pfcrt* mutant progeny also carried mutant *dmt1*; thus linkage disequilibrium may have obscured the impact of *dmt1* in *pfcrt* mutant parasites. The increase in quinine IC_50_ and IC_90_ values was observed when comparing parasites harbouring mutations in both *pfcrt* and *dmt1*, compared with parasites harbouring both WT alleles (Fig. 3a,b).

In our quinine-pressured samples, mutant *dmt1* was enriched at concentrations ≥140 nM, with 7/8 haplotypes having mutant *dmt1*, of which 5 also had mutant *pfcrt*. In contrast, without drug pressure, 9/55 haplotypes had mutant *dmt1*, of which 8 also had mutant *pfcrt* (Supplementary Fig. 1a,b and Supplementary Table 4). Collectively, the large physical separation between *pfcrt* and *dmt1* (∼289 kb), the significant *dmt1*-containing QTL obtained in WT *pfcrt* parasites, the unmasking of additional significant QTLs depending on whether *pfcrt* or *dmt1* was a covariate, and the enrichment of *dmt1* at higher quinine concentrations, support *pfcrt* and *dmt1* as two distinct peaks associated with quinine resistance.

Using ChimeraX MatchMaker, we tested for similarities between the AlphaFold3-predicted NF54 DMT1 (ref. ^41^) and the 7G8 PfCRT cryo-EM-resolved structures^42^ (Extended Data Fig. 6). The transmembrane regions showed similar folds, with a close alignment of 1.273 Å across 47 pruned atom pairs within predicted transmembrane domains. However, global structural alignment was poor (19.938 Å across all 300 atom pairs), likely reflecting low-confidence flexible regions in the AlphaFold3 model, wherein the Y107N and S129L mutations reside.

**Extended Data Fig. 7:**
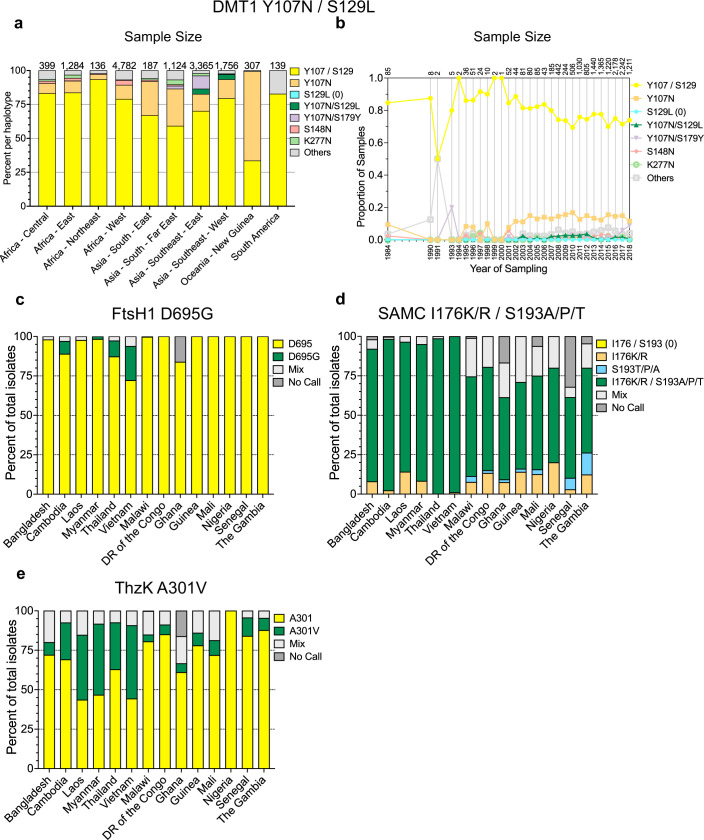
Clinical isolates harbor mutations in genes of interest within the chromosome 7 quinine and chromosome 12 quinine, chloroquine, and monodesethyl-chloroquine quantitative trait locus segments. **a–e**, Frequency of the Cam3.II mutations in clinical isolates of genes in chromosome 7 ((**a, b**), *dmt1:* PF3D7_0715800) and chromosome 12 ((**c**) *ftsh1:* PF3D7_1239700, (**d**) *samc:* PF3D7_1241600, (**e**) *thzk:* PF3D7_1239600)) loci, grouped by sampling country or year. Yellow indicates isolates with the NF54 wild-type haplotype covered by the indicated SNP positions; green, the Cam3.II mutant haplotype; light grey, mixed call; dark grey, genotype not called; orange or blue, single-amino acid mutants not corresponding to the double-mutant Cam3.II haplotype. The data to calculate (**a**) prevalence of major DMT1 haplotypes and (**b**) their change over time were obtained from the Pf7 genome database^43^ (comprising 13,479 samples). Region-specific sample sizes are shown above the plots for both panels. For **c–e**, data were compiled from the Pf3k database^64^: (*N* = 50 (Bangladesh), 570 (Cambodia), 85 (Laos), 60 (Myanmar), 148 (Thailand), 97 (Vietnam), 369 (Malawi), 113 (Democratic Republic of the Congo), 617 (Ghana), 100 (Guinea), 96 (Mali), 5 (Nigeria), 137 (Senegal), 65 (The Gambia); total = 2,512 samples). These four top candidate genes are further described in Supplementary Table 9. Source data

DMT1 sequence alignment with *Plasmodium* species revealed conserved TMHMM-predicted transmembrane domains (Supplementary Fig. 3). Analysis of 13,479 *P. falciparum* genomes from the Pf7 database^43^, spanning Asia, Africa, Oceania and South America, revealed the frequent presence of Y107N, whereas S129L was restricted to Asian isolates already harbouring Y107N. S129L was only detected from 2003 (Extended Data Fig. 7a,b), consistent with its emergence subsequent to Y107N.

*dmt1* was previously reported to be dispensable in *P. berghei*^44^ and was predicted to be potentially dispensable in cultured *P. falciparum* ABS parasites albeit with a fitness cost (Supplementary Table 9)^45^. We found zero isolates with stop codons in *dmt1* in the Pf3k genome database, suggesting essentiality in vivo.

### Mutant *dmt1* may play a modulatory role in parasite susceptibility to quinine

**Fig. 4:**
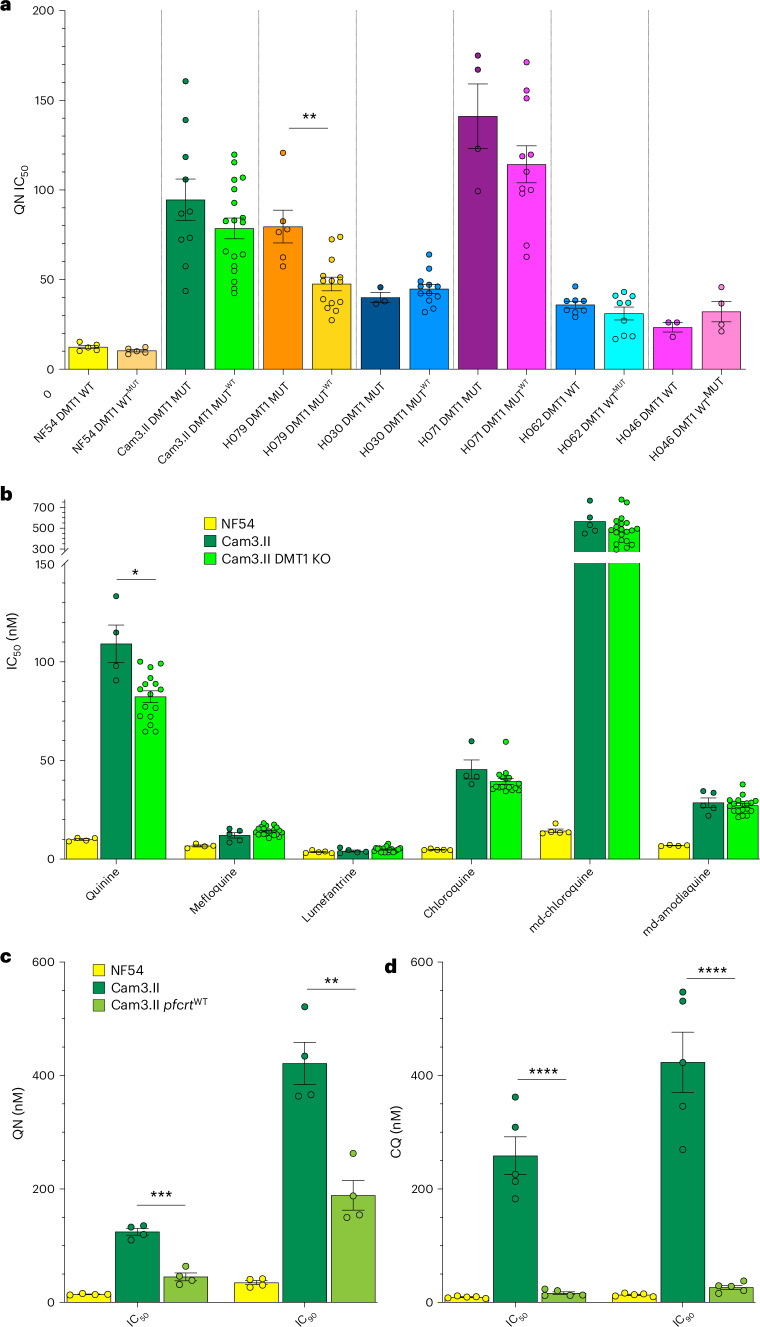
Cam3.II parasites lacking DMT1 or expressing WT PfCRT are significantly sensitized to quinine. **a**, Quinine response of DMT1 SNP-edited parents and progeny as measured by mean ± s.e.m. IC_50_ values. For each parasite, the parental unedited parasite (endogenous DMT1 haplotype) and the SNP-edited parasite (Y107N/S129L mutant or wild-type revertant haplotype) are indicated by dark and light colours, respectively. *P* values (unpaired, two-sided Student’s *t*-test, corrected with the Bonferroni–Šídák method) are indicated (*N* = 2–8 biological replicates, with technical duplicates). **b**, Quinine, mefloquine, lumefantrine, chloroquine, md-chloroquine and md-amodiaquine response in Cam3.II DMT1 knockout parasites as measured by mean ± s.e.m. IC_50_ values. *P* values (unpaired, two-sided Student’s *t*-test, corrected with the Bonferroni–Šídák method) are indicated for the Cam3.II knockout strain vs the Cam3.II parent (*N* = 4–21 biological replicates, with technical duplicates). **c**,**d**, Quinine (**c**) and chloroquine (**d**) response in Cam3.II *pfcrt*^WT^ revertant parasites as measured by mean ± s.e.m. IC_50_ and IC_90_ values. *P* values (unpaired, two-sided Student’s *t*-test) are indicated for the Cam3.II *pfcrt*^WT^ strain vs the Cam3.II parent (*N* = 4–5 biological replicates, with technical duplicates). For **a**, H079 DMT1 MUT^WT^ versus H079 DMT1 WT, *P* = 0.0011. For **b**, Quinine Cam3.II DMT1 KO versus Cam3.II, *P* = 0.0119. For **c**, Cam3.II *pfcrt*^WT^ versus Cam3.II IC_50_, *P* = 0.0001 and IC_90_, *P* = 0.0022. For **d**, Cam3.II *pfcrt*^WT^ versus Cam3.II IC_50_ and IC_90_, *P* < 0.0001. *P < 0.05, **P < 0.01, ***P < 0.001, ****P < 0.0001. IC, s.e.m. and *N* values are listed in Supplementary Table 12 and the Source data. Source data

To test whether *dmt1* modulates quinine susceptibility in vitro, we used CRISPR/Cas9 (Supplementary Fig. 4a) to edit the Y107 and S129 positions in both parents and select progeny (H079, H030, H071, H062 and H046) spanning diverse quinine susceptibilities and genotypes at *dmt1*, *pfcrt* and *ftsh1* (Supplementary Table 4). Editing was confirmed using Sanger sequencing (Supplementary Tables 10 and 11), and drug responses were compared to their unedited isogenic parasites (Fig. 4a and Supplementary Table 12).

A statistically significant sensitization to quinine was observed between the SNP-edited lines and their unedited parasites only for H079 (mutant for *pfcrt*, *dmt1* and *ftsh1*; ∼40% mean IC_50_ reduction). WGS detected no SNPs or CNVs aside from removal of the two *dmt1* mutations (Y107N/S129L) and introduction of three silent mutations to prevent re-cleavage of the edited locus. Cam3.II and H071 *dmt1* revertants (*dmt1*^WT^) showed more modest decreases (∼17% and 19% reductions, respectively). In the H062 and H046 progeny (both Dd2 *pfcrt*, WT *dmt1*), mutating *dmt1* did not alter quinine susceptibility. These data suggest that DMT1 can play a role in quinine response in a genetic background-dependent manner and evoke a potential role for additional chromosome 7 contributors.

We also profiled *dmt1* SNP-edited lines and unedited isogenic controls against chloroquine, md-chloroquine and the related 4-aminoquinoline monodesethyl-amodiaquine. These studies revealed minimal changes (Supplementary Fig. 5a–d and Supplementary Table 12), suggesting that the drug modulatory role of *dmt1* is mostly quinine specific.

### Phenotypic profiling of knockout strains supports *dmt1* as a marker of quinine resistance

To further investigate *dmt1*’s role, we generated knockout parasites in Cam3.II (Supplementary Fig. 6). Across dose–response assays (*N* = 5) with four independent *dmt1* knockout clones, the mean growth rate was calculated to be 12.7% slower than that of parental Cam3.II, which was not significantly different. Phenotypic characterization showed that the four *dmt1* knockout clones had 25–30% lower quinine IC_50_ and IC_90_ levels than Cam3.II (Fig. 4b, and Supplementary Fig. 5e and Supplementary Table 12). Knockout parasites showed increased susceptibility to mefloquine and lumefantrine at the IC_90_ but not at the IC_50_ level, evoking earlier findings of overlapping mechanisms of susceptibility to these structurally related drugs (Extended Data Fig. 1a)^13,14,46,47^.

### DMT1 localizes to the digestive vacuole, lipid bodies, parasitophorous vacuolar membrane and structures associated with vesicular trafficking

**Fig. 5:**
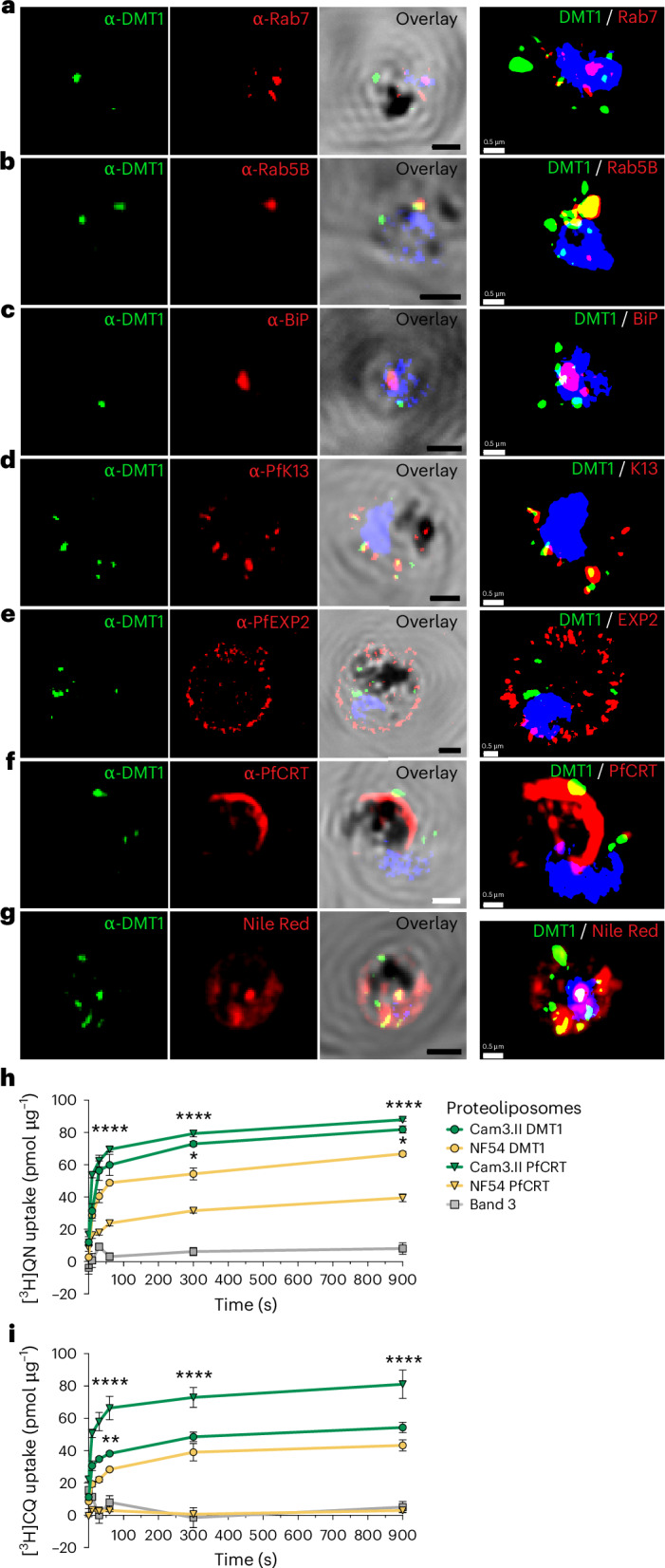
DMT1 shows localization to structures associated with vesicular trafficking as well as the digestive vacuole and exhibits differential uptake of [^3^H]quinine between the NF54 and Cam3.II isoforms. **a**–**g**, Representative immunofluorescence assays (IFAs) showing DMT1-3 × HA-tagged parasites stained with antibodies or dyes: α-HA (DMT1, green), DAPI (nuclear, blue). **a**, α-Rab7 (late endosome). **b**, α-Rab5B (early endosome). **c**, α-BiP (ER). **d**, α-PfK13 (ER, vesicles, near cytostome). **e**, α-PfEXP2 (parasitophorous vacuolar membrane). **f**, α-PfCRT (digestive vacuole membrane). **g**, Nile Red (lipid bodies). IFA images are also shown as 3D volume reconstructions (right-most column). Scale bars, 1 μm (IFA, black), 0.5 μm (3D volume reconstructions, white). DMT1-3 × HA localization was assessed in 4 independent experiments. Co-staining of DMT1 with PfEXP2 was performed in 3 independent experiments, and co-staining with BiP, Nile Red, PfCRT, PfK13, Rab5B and Rab7 was performed in 2 independent experiments. Images shown are representative of these independent experiments with similar results. **h**,**i**, Time course of 300 nM [^3^H]quinine (**h**) and 300 nM [^3^H]chloroquine (**i**) uptake measured with PfCRT (NF54 or Cam3.II), DMT1 (NF54 or Cam3.II), or Band 3-containing proteoliposomes. Data are mean ± s.e.m. of *N* = 3 independent experiments with technical duplicates. Statistical significance between NF54 and Cam3.II variants for each protein was determined using an unpaired two-sided Student’s *t*-test corrected for multiple testing using the Holm–Šídák method (**h**,**i**). *P < 0.05, **P < 0.01, ****P < 0.0001. Uptake, s.e.m., *N* and *P* values are listed in the Source data. Source data

**Extended Data Fig. 8:**
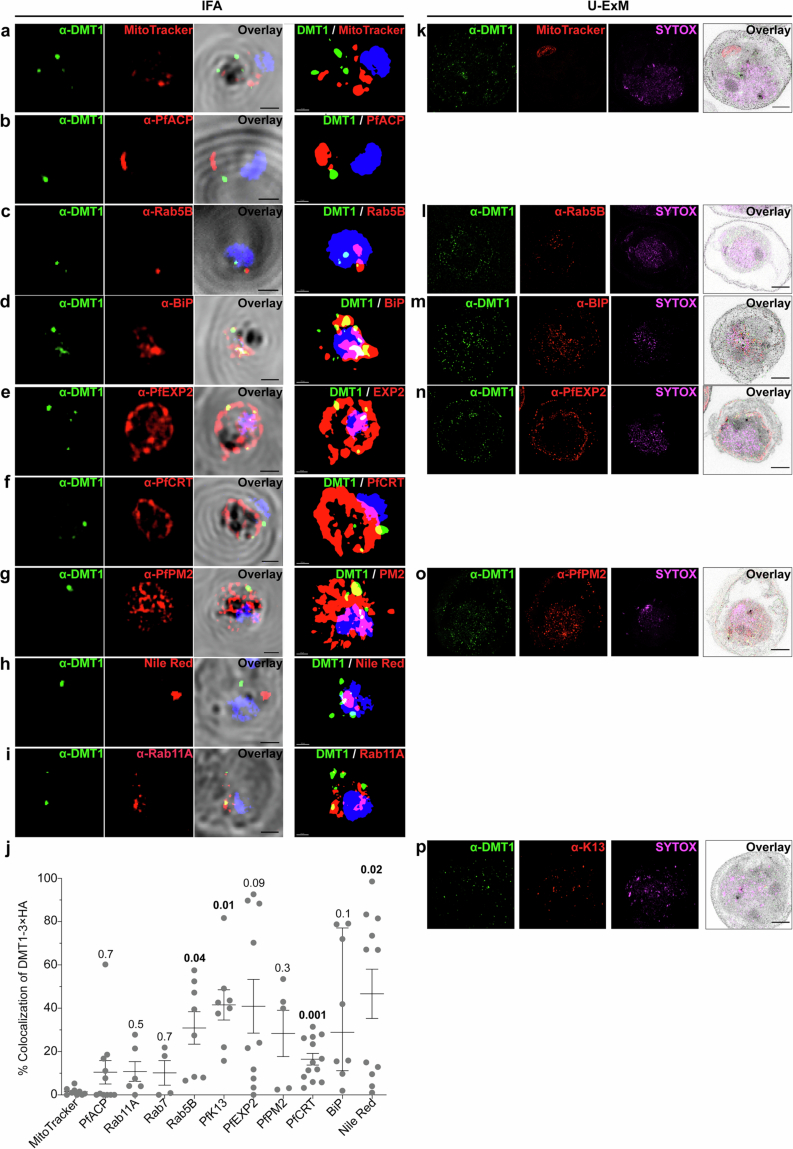
DMT1 shows localization to structures associated with vesicular trafficking as well as the digestive vacuole. **a–i**, Representative immunofluorescence assays (IFA) showing DMT1-3×HA-tagged parasites stained with *α*-HA (DMT1, green) and DAPI (nuclear, blue), as well as antibodies or dyes: (**a**) MitoTracker Red CMXRos (mitochondria, red), (**b**) *α*-PfACP (apicoplast), (**c**) *α*-Rab5B (early endosome), (**d**) *α*-BiP (ER), (**e**) *α*-PfEXP2 (parasitophorous vacuolar membrane), (**f**) *α*-PfCRT (digestive vacuole membrane), (**g**) *α*-PfPM2 (digestive vacuole), (**h**) Nile Red (lipid bodies), or (**i**) *α*-Rab11A (post-Golgi). IFA images are also shown as 3D volume reconstructions (right-most column). DMT1-3×HA localization was assessed in four independent experiments. Co-staining of DMT1 with PfEXP2, PfACP, and MitoTracker was performed in three independent experiments, and co-staining with BiP, Nile Red, PfCRT, PfPM2, Rab5B, and Rab11A was performed in two independent experiments. Scale bars: 1 μm for IFA (black) or 0.5 μm for 3D volume reconstructions (white). **j**, Mean ± s.e.m. percent DMT1-3 × HA volume colocalization between DMT1 and the aforementioned organelles (*N* = 5−13 total images). Colocalization *p* values (Welch’s ANOVA with a Dunnett’s post hoc test, both two-sided) are indicated for the % colocalization for DMT1 and the tested organelle vs colocalization between DMT1 and mitochondria (negative control). *p* values < 0.05 are indicated in bold. Numbers of individual parasites analyzed and mean ± s.e.m. and *p* values for two different colocalization metrics (volume and Manders’ coefficient) are described in Supplementary Table 13 and in the Source data. **k–p**, Representative ultrastructure expansion microscopy (U-ExM) showing DMT1-3 × HA-tagged parasites stained with *α*-HA antibodies (DMT1, green), SYTOX (nuclear, blue), NHS Ester (protein, grey) as well as antibodies or dyes specific for: (**k**) MitoTracker Red CMXRos (mitochondria, red), (**l**) Rab5B (early endosome), (**m**) BiP (ER, different antibody from IFA), (**n**) PfEXP2 (parasitophorous vacuolar membrane), (**o**) PfPM2 (digestive vacuole), and (**p**) K13 (ER, vesicles, near cytostome). U-ExM experiments were conducted twice independently. Scale bar: 5 μm for U-ExM. Source data

To explore DMT1’s subcellular localization, we generated DMT1-3 × HA-tagged NF54 ABS parasites (Supplementary Fig. 7). Immunofluorescence microscopy with anti-3 × HA antibodies revealed a punctate pattern (Fig. 5a–g and Extended Data Fig. 8a–i), whose co-localization with organelle markers and subcellular compartments was quantified (Supplementary Table 13 and Extended Data Fig. 8j).

DMT1 partially co-localized with the DV-resident markers PfCRT (mean ± s.e.m., 16.5 ± 2.7%) and plasmepsin 2 (PfPM2, 28.4 ± 10.7%) (Fig. 5f and Extended Data Fig. 8f,g). In some parasites, DMT1 almost fully (>80%) co-localized with lipid bodies (Nile Red stain), the endoplasmic reticulum (ER) and the parasitophorous vacuolar membrane (PVM) (Fig. 5c,e,g and Extended Data Fig. 8d,e,h). In other parasites, however, overlap was minimal, leading to moderate co-localization values when averaged across all parasites (46.7 ± 11.4% (lipid bodies), 39.4 ± 11.7% (ER) and 40.9 ± 12.4% (PVM)). This discrepancy may reflect DMT1 intracellular trafficking. Interestingly, Nile Red can stain DV-adjacent small cytoplasmic neutral lipid droplets that contain di- and tri-acylglycerols, which may explain the co-localization with both Nile Red and PfCRT^48–50^. DMT1 also partially co-localized with early endosomes (30.9 ± 7.5%) (Fig. 5b and Extended Data Fig. 8c), although less so with late endosomes (10.2 ± 5.7%) (Fig. 5a) or post-Golgi compartments (10.8 ± 4.5%) (Extended Data Fig. 8i). These observations suggest a role for DMT1 in vesicular trafficking. DMT1 also showed some co-localization with K13 (41.5 ± 7.0%; Fig. 5d), which is present in plasma membrane cytostomes, the ER and vesicular structures^51–53^. DMT1 did not co-localize with mitochondria or apicoplasts. Ultrastructure expansion microscopy largely recapitulated our immunofluorescence assay (IFA) findings (Extended Data Fig. 8k–p).

### Gene editing confirms a contribution of mutant PfCRT to quinine resistance

Our QTL analyses with quinine IC_50_ values revealed a chromosome 7 segment that encompasses *pfcrt*. To test this association, we reverted the *pfcrt* Dd2 allele in Cam3.II to the WT (NF54) allele, using zinc-finger nuclease-based gene editing^54^. In Cam3.II, reversion significantly reduced quinine IC_50_ and IC_90_ values (by 64% and 55%, respectively; Fig. 4c and Supplementary Table 12). Values remained higher than those of NF54, consistent with *pfcrt* being only one component of a multigenic quinine resistance trait. Reversion of mutant *pfcrt* to WT in Cam3.II fully restored chloroquine sensitivity at both IC_50_ and IC_90_ levels (Fig. 4d and Supplementary Table 12), as expected given its primary role in mediating chloroquine resistance.

### NF54 and Cam3.II DMT1 and PfCRT isoforms differentially transport [^3^H]quinine

To test whether DMT1 might transport quinine, we purified and reconstituted NF54 (WT) and Cam3.II (Y107N/S129L) DMT1 isoform proteins in proteoliposomes^42^ and measured [^3^H]quinine and [^3^H]chloroquine uptake as a transport surrogate (Fig. 5h,i). Results provided evidence that DMT1 isoforms could transport both [^3^H]quinine and [^3^H]chloroquine, with the Cam3.II isoform showing significantly higher [^3^H]quinine uptake. These data suggest that mutant DMT1 could contribute to quinine partial resistance via potential quinine transport away from its primary site(s) of action. Mutant PfCRT also transported significantly more [^3^H]quinine than its WT isoform, consistent with PfCRT also playing a role in quinine response.

For [^3^H]chloroquine, the highest level of transport was observed with Cam3.II PfCRT, whereas NF54 PfCRT showed no detectable transport, consistent with mutant PfCRT mediating chloroquine resistance via its transport away from its site of action in the parasite DV^38,55^. Lower [^3^H]chloroquine transport levels were observed with both DMT1 isoforms, consistent with mutant DMT1 not contributing to chloroquine resistance. Our data agree with previous studies partially associating mutant PfCRT with quinine resistance and observations of PfCRT transporting non-chloroquine drugs, thereby impacting parasite susceptibility by potentially changing drug concentrations at their sites of action^56^.

### Quinine partially inhibits haem detoxification

To evaluate the potential involvement of quinine and other drugs in haem detoxification, which occurs in the DV, we tested their ability to inhibit β-haematin (synthetic haemozoin) formation in a cell-free assay^57,58^. Compared with known β-haematin inhibitors chloroquine and md-amodiaquine (mean ± s.e.m. IC_50_, 11.2 ± 0.5 μM and 5.2 ± 1.0 μM, respectively), quinine and the related drug mefloquine showed weaker inhibition (22.1 ± 1.2 μM and 33.8 ± 4.6 μM, respectively), supporting their minor roles in haem detoxification^59,60^ (Extended Data Fig. 1b, and Supplementary Tables 14 and 15).

### The chromosome 12 peak harbours *samc*, *thzk* and *ftsh1* that associate with quinine and chloroquine resistance

Within the chromosome 12 QTL found with quinine, chloroquine and md-chloroquine, we identified 10 genes whose sequences differed between the parents. From these, we prioritized *samc* (PF3D7_1241600), *ftsh1* (PF3D7_1239700) and *thzk* (PF3D7_1239600) as top candidates (Supplementary Table 9), based on their previous associations with resistance to antiplasmodial compounds^61–63^. In an earlier transposon mutagenesis screen with in vitro cultured *P. falciparum* ABS parasites^45^, *samc* was predicted to be essential, whereas *ftsh1* and *thzk* were predicted to be dispensable with either no or slight fitness cost, respectively.

For these three genes, we looked for evidence of selective pressure in field isolates, using the Pf3k database^64^ (Extended Data Fig. 7c–e). FtsH1 D695G (present in Cam3.II) was observed in Southeast Asia but not in Africa. Haplotypes encoding the SAMC mutations I176K/R (K observed in Cam3.II) and S193A/P/T (A observed in Cam3.II) were prevalent, especially in Southeast Asia. The Cam3.II ThzK A301V mutation had low prevalence across Southeast Asian and African countries. These loci may not have been detected in previous chloroquine QTL analyses (HB3 × Dd2 and 7G8 × GB4 genetic crosses^16,36^) because their alleles were identical between those parents.

### FtsH1 mutations can modulate quinine and chloroquine resistance

**Fig. 6:**
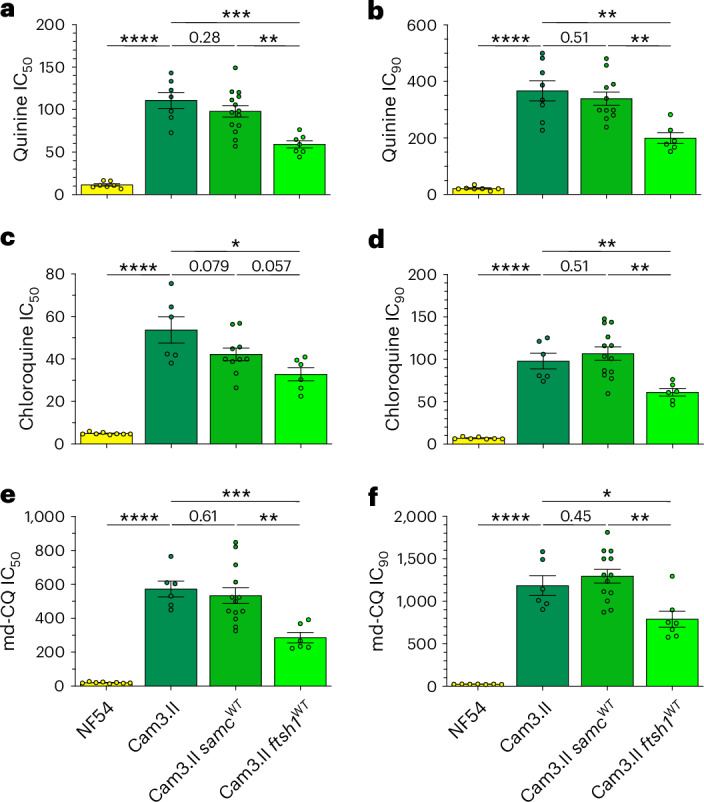
*ftsh1* is a candidate gene for the chromosome 12 QTL locus. **a**–**f**, Quinine (**a**,**b**), chloroquine (**c**,**d**) and md-chloroquine (**e**,**f**) response in *samc* and *ftsh1* SNP-edited Cam3.II as measured by mean ± s.e.m. IC_50_ or IC_90_ values. For each parasite, the parental unedited parasite (endogenous haplotype) (dark colour) and the SNP-edited parasite (wild-type revertant parasite) (light colour) were profiled. *P* values (unpaired, two-sided Student’s *t*-test) are indicated (*N* = 6–16 biological replicates, with technical duplicates). *P < 0.05, **P < 0.01, ***P < 0.001, ****P < 0.0001. IC, s.e.m., *N* and *P* values are listed in Supplementary Table 12 and the Source data. Source data

To test whether *samc, thzk* or *ftsh1* might contribute to quinine and chloroquine resistance, we reverted each mutant allele to WT in Cam3.II (Supplementary Fig. 4, and Supplementary Tables 10 and 11). Reversion of *samc* or *thzk* (denoted as *samc*^WT^ and *thzk*^WT^) showed no significant difference in susceptibility to quinine, md-chloroquine and chloroquine when compared to isogenic parental Cam3.II (Fig. 6 and Supplementary Table 12). Also, no shift was observed when introducing the *thzk* A301V mutation into NF54 (Supplementary Table 12). In contrast, reverting *ftsh1* to WT in Cam3.II significantly decreased the level of resistance to quinine, chloroquine and md-chloroquine (averaging 33–50%). These results suggest that *ftsh1* may act as a potential contributor to quinine resistance and to mutant *pfcrt*-mediated chloroquine and md-chloroquine resistance. The effect of mutant *pfcrt* on chloroquine and its metabolite is nonetheless dominant, as shown when segregating progeny on the basis of the *pfcrt* and either *thzk*/*ftsh1* (co-inherited) or *samc* alleles and comparing md-chloroquine IC_50_ values (Fig. 3c,d).

### Peptidomics analysis implicates *pfcrt*, *dmt1* and chromosome 12 loci in modulating intracellular parasite levels of haemoglobin-derived peptides

Chloroquine resistance-conferring mutant *pfcrt* is known to impact intracellular levels of haemoglobin-derived peptides, presumably via their altered efflux across the DV membrane. Given our evidence that mutant *pfcrt* contributes to quinine partial resistance, we quantified haemoglobin-derived peptides in the cross parents and select progeny carrying distinct *pfcrt*, *dmt1* and *thzk/ftsh1/samc* allelic combinations. Peptidomics analyses revealed elevated levels of haemoglobin-derived peptides in mutant *pfcrt* parasites (Supplementary Fig. 8 and Supplementary Table 16), as expected^65^. This increase was partially reversed in the H046 progeny harbouring mutant *pfcrt*, WT *dmt1* and mutant *thzk/ftsh1/samc* (Supplementary Fig. 8 and Supplementary Table 4). Our results support a dominant role for *pfcrt* in peptide homeostasis while suggesting its modulation via *dmt1* and chromosome 12 loci. Understanding the mechanistic underpinnings of these genetic interactions merits further investigation.

## Discussion

Here we identify three genetic markers associated with a polygenic basis of *P. falciparum* partial resistance to quinine, one of the oldest and most enigmatic antimalarials. Using a genetic cross between quinine-partially resistant Cam3.II and quinine-sensitive NF54 parasites, we recovered 120 unique recombinant haplotypes. QTL mapping associated quinine partial resistance with a dominant peak on chromosome 7 that included a previously uncharacterized putative drug/metabolite transporter, DMT1. A smaller upstream peak aligned with *pfcrt*. For both quinine and chloroquine, we also observed a peak on chromosome 12, including *ftsh1*. Evidence supporting a quinine resistance modulatory role for *dmt1* was obtained following SNP editing in a cross progeny and in Cam3.II knockout parasites. For *pfcrt* and *ftsh1*, SNP editing in Cam3.II resulted in quinine sensitization. These findings support assessing all three genes as candidate molecular markers for molecular surveillance of quinine partial resistance, while highlighting a complex trait dependent on the parasite genetic background.

We localized DMT1 to the DV, lipid bodies, the PVM and structures associated with vesicular trafficking. DMT1 may be trafficked by vesicular structures from the PVM to either the DV membrane or DV-adjacent lipid bodies, although we cannot exclude additional sites of action. Possibly, DMT1 may be involved in lipid storage of phospholipids that are known to promote β-haematin formation^50,66^. Structurally, DMT1 resembles PfCRT^20^. Proteoliposomes reconstituted with recombinant DMT1 protein mediated moderate levels of [^3^H]quinine uptake, with the Cam3.II variant exhibiting higher uptake than the WT protein. Mutant DMT1 might contribute to quinine partial resistance via intracellular flux of this drug away from its site(s) of action, perhaps by sequestering it. Quinine uptake was also observed with mutant PfCRT, more so than with WT PfCRT, consistent with our gene editing-based evidence that parasites expressing Cam3.II PfCRT were less sensitive to quinine than their WT PfCRT counterparts. The directionality of transport is uncertain, as quinine can, at high concentrations, inhibit haemozoin formation that occurs in the DV, but may also act on cytosolic targets^38^. DMT1 might also have a more indirect role, for example, by impacting PfCRT’s subcellular localization and/or drug transport function.

DMT1 harbours two mutations (Y107N/S129L) in Cam3.II but not in NF54. This gene was WT in previous genetic cross parents, which would explain its previous lack of detection^23,36,67,68^. In cultured parasites, *dmt1* is non-essential and Cam3.II DMT1 knockout parasites were significantly sensitized to quinine (by ~25% at the IC_50_ level), without affecting parasite susceptibility to other antimalarials (chloroquine, md-chloroquine, md-amodiaquine, mefloquine and lumefantrine). SNP-edited Cam3.II DMT1^WT^ parasites exhibited a ~17% decrease in quinine susceptibility compared to their unedited parent, and a 19–40% decrease in two progeny genetic backgrounds (H071, H079), yet none in another. The larger reduction in quinine IC_50_ levels in the Cam3.II knockout line compared with the SNP-edited line suggests that the level of DMT1 protein expression may also be important for mediating quinine resistance. Dosage effects have similarly been described for PfCRT in chloroquine resistance^65,69^. We observed no significant shift in quinine response when mutating DMT1 from WT to Y107N/S129L in three genetic backgrounds (NF54, H046 and H062), suggesting that *dmt1* is only one determinant of a multigenic basis of quinine resistance that, in a background-dependent manner, also includes mutant PfCRT. Further studies are required to edit *dmt1* mutations into additional strains, including ones with higher levels of quinine resistance (for example, >200 nM IC_50_), and to analyse whether these mutant genes operate epistatically as a complex resistance trait.

We analysed Pf7 data to determine segregation of *dmt1* and *pfcrt* alleles in field isolates (Supplementary Table 17). WT DMT1 occurred at similar frequencies with both WT (43.1%) and mutant (56.9%) PfCRT haplotypes. In contrast, Y107N DMT1 was more often associated with mutant PfCRT (64.9%), while the Cam3.II Y107N/S129L DMT1 haplotype showed a striking enrichment with mutant PfCRT (96%) (predominantly Dd2). Geographic stratification indicated that the Cam3.II Y107N/S129L DMT1–Dd2 PfCRT combination was concentrated in Southeast Asia, with S129L probably arising on the background of Y107N DMT1 + Dd2 PfCRT parasites. The high prevalence of Y107N/S129L with mutant PfCRT could be due to chance (for example, hitchhiking of Y107N/S129L in a haplotype with other beneficial drug-resistant loci that then fixed locally). Alternatively, Y107N/S129L, when combined with mutant PfCRT, could confer an (epistatic) functional advantage.

Our QTL analyses identified a previously undiscovered chromosome 12 peak that overlapped for quinine, chloroquine and md-chloroquine. Gene editing implicated *ftsh1* as a potential contributor, as reversion of the Cam3.II *ftsh1* D695G mutation to WT significantly increased susceptibility to all three drugs (Fig. 6). *ftsh1* encodes a membrane metalloprotease homologous to the bacterial membrane AAA+ metalloprotease^70^ and is required for apicoplast biogenesis in *P. falciparum* and *Toxoplasma gondii*^61^. In *P. falciparum* D10 parasites, FtsH1 G489C confers resistance to actinonin, an antibiotic that kills *P. falciparum* within a single parasite generation, unlike apicoplast ribosome-targeting antibiotics that cause delayed death^63,71^. FtsH1 mutations may confer resistance to both apicoplast- and vacuole-targeting compounds by perturbing apicoplast function, which can impair DV development as shown when apicoplast isoprenoid biosynthesis was disrupted^72^. Whether quinine might also interfere with apicoplast biogenesis remains unknown. Previous studies have localized FtsH1 to the mitochondria^70^ or apicoplast^61^, potentially reflecting distinct targeting of processed forms or close functional association between these organelles^63^. Ongoing work aims to resolve the localization and function of these processed forms.

We observed strong linkage disequilibrium between *ftsh1* and the chromosome 7 segment harbouring both *pfcrt* and *dmt1*, suggesting functional complementarity that might offset physiological defects resulting from *pfcrt* or *dmt1* mutations. A prime candidate is the excess of short globin-derived peptides in mutant *pfcrt* parasites^55,65,73,74^. When considering how FtsH1 might relate to DV transporters, we note that the apicoplast is essential for isoprenoid biosynthesis, which prenylates Rab5 proteins required for vesicle trafficking to the DV and normal DV development^72,75^.

Our study provides a rationale for testing for changing prevalences of mutant *pfcrt*, *dmt1* and *ftsh1* in areas where quinine is being used. Current quinine drug pressure is low due to its limited use only during early pregnancy and severe malaria. However, monitoring these mutations may become increasingly important if quinine use expands in response to increasing artemisinin partial resistance in Africa.

Lastly, our quinine studies highlight the value of treating malarial infections with compounds having complex modes of action that require resistance to be multigenic. Despite being the oldest antimalarial drug, quinine remains broadly effective. In future drug discovery efforts, chemical compounds with multiple *P. falciparum*-specific targets and/or multiple low-level resistance modulators merit prioritization for clinical use.

## Methods

### Parasite in vitro culture

*P. falciparum* ABS parasites were cultured in human RBCs at 3% haematocrit, using RPMI 1640 medium supplemented with 25 mM HEPES, 2.1 g l^−1^ sodium bicarbonate, 10 mg ml^−1^ gentamicin, 50 mM hypoxanthine and 0.5% (w/v) AlbuMAX II (Thermo Fisher). Parasites were maintained at 37 °C under 5% O_2_/5% CO_2_/90% N_2_ gas conditions. RBCs and human serum were procured from the Interstate Blood Bank (Memphis, TN) and RBCs were also procured from the New York Blood Center (New York, NY).

### Genetic cross

NF54 was isolated from a patient in the Netherlands in the 1970s and is presumed to be of east African origin^28^. Our parental NF54 strain was obtained from Johns Hopkins University where it has been routinely maintained in culture with human serum and retains infectivity for *Anopheles* mosquitoes. Cam3.II (also known as RF967 or PH0306-C, kindly provided by Dr Rick Fairhurst), was isolated from a malaria patient in western Cambodia in 2010 and has been classified as ART-resistant both in vivo and in vitro^24,25^. For the genetic cross, we used the G8 clone of Cam3.II, with both parents cultured in media containing 10% (v/v) human serum. NF54 and Cam3.II *P. falciparum* gametocytes were generated as previously described^29^. Briefly, parasites were propagated in RPMI 1640 media containing 10% (v/v) human serum, at 4% haematocrit and 37 °C in a glass candle jar. Cultures were initiated at 0.5% asynchronous asexual parasitaemia from a low-passage stock and maintained up to day 18 with daily media changes but without any addition of fresh RBCs. Days 15–18 cultures, containing largely mature gametocytes, were used for mosquito feeds. Cultures were centrifuged (108 × *g*, 4 min) and the parasite pellet was adjusted to 0.3% gametocytaemia in a mixture of human O+ RBCs supplemented with 50% (v/v) human serum. To conduct a genetic cross between NF54 and Cam3.II, we used a 1:1 ratio of gametocytes at a final gametocytaemia of 0.3%. Gametocytes were fed for 1 h to 4–6-day-old *Anopheles stephensi* mosquitoes (Liston strain) that had been starved overnight, using glass membrane feeders. Unfed mosquitoes were removed after feeding. Fed mosquitoes were then maintained on a 10% sugar solution at 25 °C and 80% humidity with a 14:10 h (light:dark) cycle including a 2 h dawn/dusk transition. Human RBCs used to set up the cultures were collected weekly from healthy volunteers, with informed consent, under a Johns Hopkins University Institutional Review Board approved protocol (number NA_00019050). All experiments were performed in accordance with institutional guidelines and regulations.

On day 7 post-infected blood meal, mosquito midguts were collected and stained with mercurochrome. Oocysts were counted by brightfield microscopy using a Nikon E600 microscope with a PlanApo ×10 objective. We observed a ~85% prevalence of infection, with an average of ~20 oocysts per mosquito. Between days 14 and 16, salivary glands were collected from ~35 mosquitoes, homogenized and the sporozoites counted using a haemocytometer. On average, we obtained 40,000 salivary gland sporozoites per mosquito.

All animal experiments were performed in accordance with the Animal Care and Use Committee (ACUC) guidelines and approved by the Johns Hopkins ACUC (Protocol M017H325), with modifications to a protocol reported previously^76^. Female (6–7-month-old) FRG NOD human liver-chimaeric (huHep) mice^77^ were purchased and shipped from Yecuris Corporation. Upon arrival, we began withdrawal of the mice from 2-(2-nitro-4-trifluoromethylbenzoyl)-1,3-cyclohexanedione (NTBC). Mice were housed in a sterile facility with sterile bedding, food and water. Mice were weighed daily and those that showed weight loss >10% (compared to pre-shipment weight) were treated with 300 μl of sterile saline via intraperitoneal injection and given a nutritional supplement in water (STAT; https://www.prnpharmacal.com/products/nutritional-supplements/stat/). Before infection, mice were allowed to recover from shipping for 2–3 days. Infection with *P. falciparum* sporozoites was performed either by mosquito bite or by intravenous (i.v.) inoculation. For the mosquito bite approach, mosquitoes were allowed to probe/feed for 17–19 min on mice anaesthetized with 150 μl ketamine/xylazine. Two mice (A and B) each received mosquito bites from ~60 mosquitoes. Two other mice (C and D) were given prophylactic penicillin/streptomycin antibiotics and then i.v. inoculated with 200,000 sporozoites dissected from mosquito salivary glands. Mice infected by mosquito bite remained stable and healthy appearing for the remainder of the experiment. In contrast, both mice infected by i.v. inoculation lost ~5% of their body weight relative to their pre-shipment weight and were given extra rehydration gel food. On days 5.5 and 6.5 post-sporozoite infection, 450–500 μl of RPMI-washed human RBCs at ~70% haematocrit were i.v. inoculated into each mouse (except for an intraperitoneal injection for Mouse B due to scar tissue at the tail vein injection site). Mice were given new dextrose, cages and rehydration gel food on both days. At day 7 post infection, ABS parasites were detected at 0.3–0.8% parasitaemia and blood was recovered by cardiac puncture of mice anaesthetized with ketamine/xylazine (Supplementary Table 1). Bulk freezes were made after one to two ABS cycles in culture. The bulk freezes made after one cycle were thawed from each mouse and used to obtain progeny clones by limiting dilution cloning (referred to as clones from ‘no drug pressure’ before cloning). The post-two-cycle freezes for mice B and C were later thawed and pressured at 50 nM or 75 nM chloroquine, or 75 nM or 95 nM quinine, for 3 days in 5 ml cultures at 3% haematocrit. Surviving parasites were then cloned and cryopreserved. Mouse B ‘no drug’, as well as mouse B and C quinine 95 nM pre-selected bulks, were thawed and pressured at quinine 0 nM, 140 nM (and later 240 nM), or 180 nM (and later 240 nM) for 4 days for bulk selection experiments. Drug-pressured bulks were cloned by limiting dilution and cryopreserved. Clones were then adapted to media devoid of 10% human serum, and all phenotyping was conducted in serum-free Albumax media.

### Genomic DNA extraction

Parasite genomic DNA (gDNA) was obtained from cultures at 5–10% parasitemia with mostly trophozoite and schizont stages. RBCs were lysed with 0.1% saponin in 1× PBS and parasite DNA extracted with the QIAamp DNA Blood midi kit (Qiagen) using a combined RNase and Proteinase K treatment. DNA concentrations were determined using a NanoDrop and/or the Qubit dsDNA BR assay kit (Thermo Fisher).

### WGS of genetic cross progeny pools and clones

Parasite samples were subjected to WGS using the Illumina Nextera DNA Flex library preparation protocol. Briefly, 150 ng of gDNA was fragmented and tagmented using bead-linked transposons and subsequently amplified by five cycles of PCR to add dual-index adapter sequences to the DNA fragments. The libraries were quantified, pooled and sequenced on either an Illumina MiSeq system using the MiSeq Reagent Kit v.3 (paired-end 300-bp reads) or a NextSeq 550 system using the High Output kit (paired-end 150-bp reads).

Progeny were sequenced either directly (for bulk pools and clones after quinine 140 nM, 180 nM and 240 nM pressure) or after clonal recombinants had been identified by microsatellite and SNP genotyping (for clones following no drug pressure or chloroquine 50 nM, chloroquine 75 nM, quinine 75 nM or quinine 95 nM pressure). The WGS data for the parents and progeny clones are visualized in Fig. 1a. Sequence data were aligned to the *P. falciparum* 3D7 genome (PlasmoDB v.48) using Burrows–Wheeler Alignment (BWA)^78^. Reads that did not map to the reference genome and PCR duplicates were removed using SAMtools and Picard. The reads were realigned around indels using the Genome Analyses Tool Kit (GATK) RealignerTargetCreator, and base quality scores were recalibrated using GATK BaseRecalibrator.

Using SAMtools mpileup, variants were called and filtered on the basis of quality scores (minimum base quality score ≥20, mapping quality >30, read depth ≥5) and multiallelic = FALSE to obtain high-quality SNPs that were annotated with SnpEff^79^. SNPs for highly polymorphic surface antigens and multigene families were removed. Homozygous SNPs that differed between the NF54 and Cam3.II parents were retained, defined as having >90% alternate or reference allele frequency for Cam3.II or NF54, respectively. We then selected one recombinant progeny per haplotype with the best sequencing coverage (lowest % of SNPs classified as heterozygous (allele frequency of >10% and <90%) or missing (no reads or reads that did not pass the variant caller’s quality filter, or DP < 5)) to create a list of 120 haplotype genomes, alongside the parents. We further culled these loci if >90% of the 120 progeny had either NF54 or Cam3.II calls and were not multi-allelic. We also removed 48 SNPs that were classified as heterozygous or discrepant for NF54 and/or Cam3.II across multiple sequencing runs. This process led us to retain 13,116 high-quality SNPs that differed between NF54 and Cam3.II. With the JoinMap v.5 (Kyazma) software, 1,836 of these SNPs were used to construct a genetic map and calculate the recombination rate.

Microsatellite sizes in the NF54 and Cam3.II parental strains were identified as described previously^80^. Briefly, to determine the confidence of a microsatellite on the basis of the number of reads, we quantified reads that harboured insertions or deletions at specified genomic loci within the respective windows^81^. Integrative Genomics Viewer was used to verify the data.

### Identification of unique recombinants

PCR and gel electrophoresis microsatellite genotyping (*N* = 11 markers; Supplementary Table 18) was used to narrow down which of the 175 progeny obtained after no drug pressure were recombinant clones, before their gDNA was whole-genome sequenced. This work yielded 162 clonal progeny (listed in Supplementary Tables 19 and 20), of which 71 were chosen for WGS analysis. To obtain additional unique recombinants, we also subjected bulk progeny pools from mice B and C to various chloroquine and quinine drug pressures (Extended Data Fig. 2b and Supplementary Table 19). We applied chloroquine 50 nM, chloroquine 75 nM, quinine 75 nM or quinine 95 nM for 3 days, while we applied quinine 140 nM (and later 240 nM), and quinine 180 nM for 4 days on the ‘no drug’ bulks or the quinine 95 nM pre-pressured bulks. After a drug wash-off and a recovery period, we obtained 133 clonal progeny from chloroquine pressure and an additional 292 from quinine pressure. Out of 303 progeny obtained after chloroquine 50 nM, 75 nM and quinine 75 nM or 95 nM selections, 296 were genotyped with 9 markers: 2 sets of 3 microsatellite markers via multiplexed capillary electrophoresis-based genotyping (Supplementary Table 18), as well as SNPs in *pfcrt* (A220S), *dhfr* (N51) and *pfmdr1* (Y184F). These 9 markers were used to screen for clonal recombinant progeny before a subset of 92 progeny were whole-genome sequenced. As the primary goal of the higher quinine concentrations was to recover quinine-resistant progeny, we split cloning plates 1:2 for progeny obtained after quinine 140 nM, 180 nM or 140 + 240 nM, and subjected plates to 72 h of 140 nM quinine. Surviving resistant parasites then proceeded to WGS analysis for 95 out of 160 progeny. PCR and either fragment analysis or gel electrophoresis-based microsatellite genotyping always correctly predicted the self-fertilized or recombinant status of the progeny, which was also confirmed by WGS.

To identify which progeny were isogenic and to assign unique haplotypes, WGS results for the 13,116 high-quality SNP positions were hierarchically clustered for the parents and clonal progeny by average linkage using Gene Cluster (3.0)^82^. The resulting dendrograms were visualized using Java TreeView^83^. The SNP inputs were as follows: NF54 allele = −3, Cam3.II allele = 3, heterozygous or missing = 0. Clustering of the unique recombinant haplotypes and the two parents were also conducted. If isogenic progeny shared the same haplotype, then the sequence for the progeny with the best sequencing data (lowest percentage of mixed and missing SNP calls) was used as the representative haplotype for subsequent clustering analysis.

### Parasite drug-susceptibility assays

Parasites were synchronized for at least two consecutive ABS cycles before starting drug-susceptibility assays and at least once a week thereafter, by exposing ABS cultures to 5% D-sorbitol (Sigma) for 15–20 min at 37 °C to remove mature parasites, followed by a wash step. In drug-susceptibility assays, predominantly ring-stage parasites were exposed to a serially diluted range of drug concentrations (2-fold, 10-point dilutions for all drugs except for quinine that used 12-point dilutions), or 700 nM dihydroartemisinin (kill control), or a no drug control. Assays started at 0.2% parasitaemia and 1% haematocrit in 96-well flat-bottom plates incubated at 37 °C for 72 h in 5% O_2_/5% CO_2_/90% N_2_. Final parasitaemias were measured using an IntelliCyt iQue Screener Plus cytometer (Sartorius), a BD Accuri C6 Plus cytometer with a HyperCyt plate sampling attachment (IntelliCyt), or a BD FACSCelesta flow cytometer with the BD High Throughput Sampler (HTS). Cells were stained with 1× SYBR Green I (Invitrogen) and 100 nM MitoTracker DeepRed FM (Invitrogen) for at least 20 min and diluted in 1× PBS before sampling (typically ~8,000–10,000 RBCs per well). Parasitaemia in each well was determined as the percentage of MitoTracker-positive SYBR Green I-positive infected RBCs within the cell subpopulation gated in the FSC versus SSC plot. The flow cytometry gating strategy is exemplified in Supplementary Fig. 9. Parasitaemia was then normalized by subtracting the background parasitaemia in the 700 nM dihydroartemisinin kill control, and IC_50_ and IC_90_ values were calculated by nonlinear regression analysis (Prism v.10, GraphPad). For that analysis, we used a 4-parameter curve fitting where minimum and maximum parasitaemias were set to 0% and 100% growth, respectively. Drug-susceptibility assays were performed with quinine, chloroquine, md-chloroquine, md-amodiaquine, lumefantrine and mefloquine. To estimate mean growth rates, for each assay, parasitaemia in the absence of drug was divided by the starting parasitaemia fixed at 0.2%. The data were then averaged across all assays. Statistical significance was determined using non-parametric, two-tailed Mann–Whitney *U*-test for quinine, chloroquine and md-chloroquine QTLs, and Student’s *t*-test for all other drug assays (Prism v.10, GraphPad). We note that in 72 h dose–response assays (*N* = 49) beginning with synchronized ring stages, Cam3.II propagated 11.4% faster than NF54.

### Quinine bulk segregant analysis

Recombinant bulk pools from Mouse B and C (with or without previous quinine 95 nM drug pressure) for the NF54 × Cam3.II cross were used for quinine bulk segregant analysis (Extended Data Fig. 2d; full sample list detailed in Supplementary Table 5). Bulk pools were pressured for 4 consecutive days in a 5 ml culture at 3% haematocrit in culture media (as mentioned above) supplemented with 10% (v/v) human serum, starting at 1% total parasitaemia. gDNA, used for library preparations and WGS, was collected before (day 0 control) and after the 4-day selections (changing media every day with drug wash-off after 4 days) at 0 nM, 140 nM, 180 nM and 240 nM quinine, and later (day 4+) when there was sufficient genomic material to collect (∼2% parasitaemia). The 0 nM no drug control well was set up at the same time as each drug selection, to ensure drug-specific killing of the pressured well and to collect no drug control gDNA at the same timepoint as its corresponding drug-pressured well. This ‘no drug timepoint’ control was used to account for allele frequency changes due to in vitro culture alone.

The gDNA from the 33 bulk pool samples (Supplementary Table 5) was whole-genome sequenced and used for the quinine bulk segregant analysis (Extended Data Fig. 4) performed with the R package, QTLseqr^84^. As some WGS data from gDNA bulk cultures exhibited low coverage compared to the progeny clones, we used all 106,316 SNPs that distinguished the two parents. For each pairwise comparison between control and quinine-pressured bulks, SNPs were then filtered using the filterSNPs function^84^ with the following restrictions: 0.1 ≤ reference allele frequency ≤ 0.9 to remove over- and underrepresented SNPs in both bulks, (minimum total depth = 10)/(maximum total depth = 200) and minimum sample depth = 5 to filter extremely low- and high-coverage SNPs in both bulks and in each bulk separately, respectively. SNPs were also removed if there was ≥100 read depth difference between bulks. QTLs were identified by comparing quinine-pressured bulks with control bulks using the QTL-seq and G’ approaches^85,86^. A window size of 100 kb was used to calculate the tricube-smoothed ∆(SNP-index) and G statistic (or G’) for each SNP within that window. For the G’ analysis, outlier regions were filtered on the basis of Hampel’s rule, and QTLs were identified as regions with false discovery rate (Benjamini–Hochberg-adjusted *P* values or *q*-values) <0.01. For the ∆(SNP-index) analysis, QTLs were identified as regions that pass the 95% and 99% confidence intervals.

### Progeny clone-based QTL mapping

The R package, R/qtl (v.2)^87^, was used to map QTL peaks (Fig. 2 and Extended Data Fig. 5b,c), using progeny IC_50_ and IC_90_ values as ‘phenotypes’ (Fig. 1b–d, Extended Data Fig. 5a and Supplementary Table 4) and their WGS data as ‘genotypes’. To identify significant QTLs for each drug response phenotype, 1,000 permutations of phenotypic data (geometric mean IC_50_, IC_90_) were performed to obtain a distribution of maximum LOD scores. These LOD scores were then used to calculate the LOD threshold at 95% probability. Fine mapping of the QTL segments was performed using Bayesian interval mapping at a 95% confidence level. Significant QTL segments are listed in Supplementary Table 7, and the genes within these segments are shown in Supplementary Table 8.

### DMT1 sequence alignment

The DMT1 protein sequence for the *P. falciparum* NF54 (PF3D7_0715800) strain and the orthologous DMT1 sequences from reference strains of *P. reichenowi* (PRCDC_0713100), *P. adleri* (PADL01_0713800), *P. gaboni* (PGSY75_0715800), *P. vivax* (PVP01_1424900), *P. knowlesi* (PKNH_1424800), *P. ovale* (PocGH01_14032100), *P. malariae* (PmUG01_14040800), *P. cynomolgi* (PcyM_1426000), *P. chabaudi* (PCHAS_1423900), *P. berghei* (PBANKA_1422100), *P. yoelii* (PY17X_1424100) and *P. gallinaceum* (PGAL8A_00520500) were obtained from the PlasmoDB.org database^88^. Clustal Omega^89^, which uses the HHalign algorithm, was used to perform multiple sequence alignments and generate phylogeny trees (Supplementary Fig. 3). EMBOSS Needle, which uses the Needleman–Wunsch algorithm, was used to perform global pairwise sequence alignments and calculate the percentage of positions in the alignment that are identical^90^. Both bioinformatic tools were accessed through EMBL-EBI^91^.

### Gene editing using CRISPR/Cas9 plasmids

The DMT1 (PF3D7_0715800) Y107N/S129L mutation or WT with binding-site mutations was introduced into the cross parents (NF54, Cam3.II) and five progeny (B-QN95-140-A5, B-CQ75-1-H9, B4-5, B5-4, Clow6, C-QN95-180-F8) using an all-in-one CRISPR/Cas9 plasmid (pDC2-co*Sp*Cas9-h*dhfr*^92^) (Supplementary Fig. 4). The *P. falciparum* codon-optimized Cas9 endonuclease was derived from *Streptococcus pyogenes* and was expressed under a *calmodulin* promoter. The plasmid also carried a human dihydrofolate reductase (h*dhfr*) selectable marker (conferring resistance to the antifolate WR99210) under a *P. chabaudi dhfr*-*ts* (*Pcdt*) promoter and a *U6* promoter for expressing the guide RNA (gRNA). The DMT1 WT and Y107N/S129L donors were synthesized in a pUC-GW-*amp* vector (Genewiz), with both donors also containing the same three silent (synonymous) mutations at the gRNA recognition site (referred to herein as binding-site mutations or bsmut). These mutations prioritized the PAM and/or the ‘seed’ region <12 nt upstream of the PAM to prevent Cas9 endonuclease-mediated re-cleavage of the donor or the edited recombinant locus. Donors were PCR amplified from the pUC-GW-*amp* vector and subcloned by in-fusion cloning into pDC2-co*Sp*Cas9-h*dhfr* at the *Eco*RI and *Aat*II restriction sites. A gRNA was selected using ChopChop (v.3)^93^ and Benchling (https://www.benchling.com), as close as possible to the mutation site, avoiding poly-T stretches of >3 Ts as they may cause premature transcription termination^94^, and prioritizing gRNAs with low off-target scores and at least 3–4 mismatches to other sites in the genome that are preferably in the PAM and/or the ‘seed’ region. Double-stranded gRNA blocks were synthesized as single-stranded DNA (Eurofins Genomics) and then phosphorylated, annealed and cloned into the *Bbs*I restriction sites of pDC2-co*Sp*Cas9-h*dhfr* by T4 DNA ligase cloning.

CRISPR/Cas9 was also used to generate Cam3.II parasites with WT ThzK (PF3D7_1239600) or the A301V mutant, WT FtsH1 (PF3D7_1239700) or the D695G mutant, and WT SAMC (PF3D7_1241600) or the I176K/S193A variant. Donors were synthesized in the pUC-GW-*amp* vector as described above, with synonymous binding-site (bsmut) mutations introduced for each gene for each of two separate gRNAs. The donors for ThzK and FtsH1 were subcloned into pDC2-co*Sp*Cas9-h*dhfr* as described above, while the donor for SAMC was kept in the pUC-GW-*amp* vector and co-transfected instead due to the low complexity of *samc*. Two gRNAs per gene were cloned into the *Bbs*I restriction sites to generate two separate plasmids for each gene, as described above.

To generate a PfNF54 DMT1 3′ 3 × human influenza haemagglutinin-based (HA) tagged parasite (Supplementary Fig. 7), a DMT1 donor with a homology region (HR) 1 (342 bp), recodonized 3′ end of DMT1 to disrupt homology with no stop codon (165 bp), spacer (6 bp), 3 × HA tag with a stop codon, and 3′ untranslated region (UTR) HR2 (509 bp) was synthesized in a pUC-GW-*amp* vector (Genewiz), PCR amplified and cloned by in-fusion cloning into the *Eco*RI/*Aat*II restriction sites of the pDC2-co*Sp*Cas9-h*dhfr* vector. Three gRNAs within the recodonized region were selected and cloned into the vector.

To generate a Cam3.II DMT1 knockout using CRISPR/Cas9 gene editing (Supplementary Fig. 6), 1,202 bp out of the 1,305-bp-long native *dmt1* exon was replaced with a selectable marker cassette expressing h*dhfr* under the control of the *PcDT* promoter and a 3′ UTR from *hrp2*. Human *dhfr* expression was selected using 2.5 nM WR99210. The left homology (499 bp) and right homology (437 bp) regions were PCR amplified from Cam3.II gDNA and inserted by in-fusion cloning into the *Eco*RI/*Aat*II and *Apa*I restriction sites of the pDC2-co*Sp*Cas9-h*dhfr* vector, respectively. Three gRNAs within the deleted *dmt1* sequence were selected and cloned into the vector.

All final plasmids were purified using the NucleoBond Xtra Maxi kit (Macherey-Nagel) and confirmed by restriction digests and Sanger sequencing (visualized using SeqMan Ultra v.17.4.1 (DNASTAR)), and are listed in Supplementary Table 11. Primers used for cloning and verification are described in Supplementary Table 10.

### Parasite transfections

ABS parasites were electroporated with purified circular plasmid DNA as previously described^95^. Per transfection, a 2.5 ml culture of 3% haematocrit, >5% ring-stage parasites was collected and washed with 15 ml of 1× Cytomix. A volume of 75 μl of parasitized RBCs was then added to 50 μg of plasmid DNA and 1× Cytomix to a total volume of 440 μl, transferred to a 0.2-cm cuvette (Bio-Rad), and electroporated at a voltage of 0.31 kV and capacitance of 950 μF using a Gene-Pulser (Bio-Rad)^96^. For SAMC SNP editing, we co-transfected 50 μg each of pUC-GW with donor and CRISPR/Cas9 plasmid with one gRNA; for DMT1 knockout, DMT1-3 × HA, FtsH1 SNP and ThzK SNP gene editing, we co-transfected 50 μg each of CRISPR/Cas9 all-in-one plasmids that contained the same donor but two distinct gRNAs. Electroporated cells were transferred from the cuvette to a well in a 6-well plate containing 3% haematocrit blood + media mixture, and media were generally changed ∼3–4 h post transfection (or sometimes ∼15 h later). Starting the day after the transfections, the cultures were maintained in 2.5 nM WR99210 (if parasites have triple *dhfr* mutations: N51I/C59R/S108N) or in 1 nM WR99210 (if WT *dhfr*) for 6 consecutive days^97^. WR99210 was procured from Jacobus Pharmaceuticals. Successful gene editing was assessed via Sanger sequencing of PCR products amplified directly from bulk cultures or from bulk culture gDNA. DMT1-3 × HA-tagged parasite clones were obtained by limiting dilution. Successful *dmt1*-3 × HA tagging was confirmed via PCR, Sanger sequencing, immunofluorescence and western blot assays. Successful *dmt1* knockout was confirmed by PCR, Sanger sequencing and gel electrophoresis. Oligonucleotide primers used in this study are listed in Supplementary Table 10.

### Western blot confirmation of DMT1-3 × HA tagging

Western blots were performed with lysates from NF54 DMT1-3 × HA-tagged parasites and NF54 PRELI-3 × HA-tagged parasites (positive control) (Supplementary Fig. 7b). Parasite cultures were washed twice in cold 1× PBS, and parasites were isolated by treatment with 0.05% saponin in PBS. Released parasites were lysed in 4% SDS, 0.5% Triton X-100 and 0.5% PBS supplemented with 1× protease inhibitors (Halt Protease and Phosphatase Inhibitor Cocktail, Thermo Scientific) and Pierce Universal Nuclease for Cell Lysis (Thermo Scientific). Samples were centrifuged at 20,000 × *g* for 10 min to pellet cellular debris. Laemmli Sample Buffer (4×, Bio-Rad) was added to lysates and samples were denatured at 50 °C for 15 min (without boiling to prevent aggregation of the DMT1 transmembrane protein). Proteins were electrophoresed on precast 4–20% Tris-glycine gels (Bio-Rad) and transferred onto nitrocellulose membranes. Western blots were probed with a 1:1,000 dilution of primary antibodies to HA (mouse mAb; Cell Signaling, 2367S, Clone 6E2), followed by a 1:5,000 dilution of anti-mouse IgG H&L HRP-conjugated secondary antibodies (Abcam, ab6789). Western blots were revealed using Pierce ECL Western Blotting Substrate (Thermo Scientific) and imaged on a ChemiDoc system (Bio-Rad).

### Indirect IFAs

Collected NF54 DMT1-3 × HA-parasitized RBCs were washed twice in 1× PBS and fixed in methanol-free 4% (v/v) formaldehyde (Pierce), supplemented with 0.0075% (v/v) glutaraldehyde (Electron Microscopy Sciences) in 1× PBS for 30 min at room temperature while stationary, followed by one 1× PBS wash. For mitochondria staining, collected parasites were washed once with pre-warmed RPMI, stained with 50 nM MitoTracker Red CMXRos (Invitrogen) at 37 °C in the incubator while stationary before the 1× PBS wash and fixation, and were kept covered throughout. Cell membranes were then permeabilized in 0.1% Triton X-100 in 1× PBS for 30 min at room temperature while stationary, followed by three 1× PBS washes. Autofluorescence was quenched using 0.1 M glycine in 1× PBS for 15 min at room temperature while rotating. Blocking was performed with 3% (w/v) bovine serum albumin (BSA) and 0.1% Tween-20 (v/v) in 1× PBS overnight at 4 °C while rotating. Cells were incubated with primary antibodies for 2–4 h at room temperature while rotating, with dilutions ranging from 1:50 to 1:200, followed by a 1-h incubation with a species-specific fluorophore-conjugated secondary antibody diluted in 3% BSA and 0.1% Tween-20 in 1× PBS. As primary antibodies, we used rabbit anti-binding immunoglobin protein (BiP; 1:200; kindly provided by Min Zhang), mouse anti-PfK13 (1:100; clone E3 (ref. ^52^)), rabbit anti-Rab11A, rat anti-Rab5B or -Rab7 (1:50; kindly provided by Dr Gordon Langsley), rabbit anti-PfACP (1:200; kindly provided by Dr Geoff McFadden), Nile Red (2.5 μg ml^−1^; Invitrogen), rabbit anti-PfEXP2 (1:200; MR4), mouse anti-PfCRT (1:200; clone 2 (ref. ^98^)) and rabbit anti-plasmepsin 2 (PM2) (1:100; BEI Resources, MRA-66). For primary antibodies raised in rabbits, we used Alexa Fluor Plus 594-conjugated goat anti-rabbit IgG (H + L) secondary antibody (1:3,000; Invitrogen, A32740). For mouse primary antibodies, Alexa Fluor 594-conjugated goat anti-mouse secondary antibodies (1:2,000; Invitrogen, A-11005) were used. For assays with rat primary antibodies, we used Alexa Fluor 594-conjugated goat anti-rat secondary antibody (1:2,000; Invitrogen, A-11007). Rat anti-HA (1:2,000; Millipore Sigma, 11867423001, Clone 3F10) and Alexa Fluor 488-conjugated goat anti-rat secondary (1:2,000; Invitrogen, A-11006) antibodies were used to test for DMT1-3 × HA expression. Samples were also co-stained with MitoTracker Red CMXRos, Nile Red or primary antibodies raised in mice or rabbits. Mouse anti-HA (1:100; Cell Signaling, 2367S, Clone 6E2) and Alexa Fluor 488-conjugated goat anti-mouse secondary (1:1,000; Invitrogen, A-21121) antibodies were used to probe for DMT1-3 × HA in co-stains with primary antibodies raised in rats.

Thin blood smears of stained RBCs were prepared on microscope slides and mounted with high-performance/high-tolerance coverslips (Zeiss) using ProLong Diamond Antifade Mountant with DAPI (Invitrogen) pre-warmed to room temperature. Mounted samples were cured overnight and imaged using a Nikon TiE Eclipse inverted microscope with a Nikon A1 scanning confocal with a GaAsP spectral detector and a CFI Plan Apochromat Lambda oil immersion objective with ×100 magnification (1.45 numerical aperture). *Z*-stacks (0.2 μm step size) were taken for each parasitized RBC. NIS-Elements v.5.02 (Nikon) was used to control the microscope and camera and crop images. Imaris v.9 (Oxford Instruments) was used to perform deconvolution using 5 (for DAPI) or 10 (for HA tags and parasite proteins) iterations of the Richardson–Lucy algorithm for each image and quantify co-localization of the deconvolved *Z*-stacks using the co-localization module. Trophozoite-stage parasites were used to quantify co-localization of DMT1 with organelles, as there was more spatial distribution of organelles than in rings, and trophozoites were less prone to variability than the multinucleated schizonts. Statistical analyses were performed with Prism v.10 (GraphPad). Fiji (v.2.11.0)^99^ (ImageJ) was used to adjust brightness and contrast, overlay channels and prepare montages. For 3D rendering of the deconvolved images, we used the Imaris software.

### Ultrastructure expansion microscopy

Expansion microscopy was performed as previously published^100^. Briefly, NF54 DMT1-3 × HA-tagged parasites (at least 5% parasitaemia) were collected. For mitochondria staining, collected parasites were washed once with pre-warmed RPMI, stained with MitoTracker Red CMXRos (50 nM; Invitrogen) at 37 °C for 30 min, before a 1× PBS wash and subsequent microscopy preparation steps outlined below, and were kept covered throughout. Parasite culture was diluted to 0.5% haematocrit and transferred onto poly-D-lysine-coated 12 mm round coverslips (Fisher Scientific) in 24-well plates. Plates were incubated at 37 °C for 30 min and samples were fixed with 4% v/v formaldehyde in PBS for 20 min at 37 °C. Samples were washed with PBS and incubated in anchoring solution (1.4% v/v formaldehyde, 2% v/v acrylamide in PBS) overnight at 37 °C. Coverslips were washed once with 1× PBS, and gelation was done by incubating coverslips with parasites facing down with 35 μl of monomer solution (19% w/w sodium acrylate, 10% v/v acrylamide, 0.1% v/v *N*,*N*’-methylenbisacrylamide in PBS supplemented with fresh 0.5% w/v ammonium persulfate (APS) and 0.5% v/v tetramethylethylendiamine (TEMED)) on a parafilm in a pre-cooled chamber for 5 min, then for 1 h at 37 °C, protected from light. Coverslips were transferred to 6-well plates and incubated in denaturation buffer (200 mM SDS, 200 mM NaCl, 50 mM Tris pH 9.0 in Milli-Q H_2_O) for 20 min at room temperature. Gels were gently removed from the coverslips, transferred to 1.5 ml tubes containing fresh denaturation buffer and heated at 95 °C for 90 min. The samples were cooled, transferred to Petri dishes and expanded in Milli-Q H_2_O at room temperature for 30 min on a shaker and the process was repeated twice.

For staining, the gels were shrunk by washing with 1× PBS for 15 min at room temperature, transferred to 6-well plates and blocked with 3% w/v BSA/PBS for 30 min at room temperature. Primary antibodies diluted in 3% BSA in 1× PBS were added to each well overnight at room temperature with gentle shaking. Gels were washed with 0.5% v/v Tween-20 in PBS three times for 10 min each at room temperature and then probed with secondary antibodies or dyes diluted in 1× PBS for 2.5 h at room temperature with gentle shaking, protected from light. Gels were washed with 0.5% v/v Tween-20 in 1× PBS as previously described and transferred to Petri dishes containing Milli-Q H_2_O for the second round of expansion. Gels were washed twice with Milli-Q H_2_O at room temperature for 30 min each and stored at 4 °C before imaging. As primary antibodies, we used rabbit anti-binding immunoglobin protein (BiP; 1:500; kindly provided by Dr Jeffrey Dvorin and distinct from the BiP antibody used for IFAs), mouse anti-PfK13 (1:250; clone F10 (ref. ^52^)), rat anti-Rab5B (1:250; kindly provided by Dr Gordon Langsley), rabbit anti-PfEXP2 (1:500; MR4) and rabbit anti-plasmepsin 2 (PM2) (1:500; BEI Resources, MRA-66). For primary antibodies raised in rabbits, we used Alexa Fluor Plus 594-conjugated goat anti-rabbit IgG (H + L) secondary antibodies (1:500; Invitrogen, A32740). For mouse primary antibodies, Alexa Fluor 594-conjugated goat anti-mouse secondary antibodies (1:500; Invitrogen, A-11032) were used. For assays with rat primary antibodies, we used Alexa Fluor 594-conjugated goat anti-rat secondary antibodies (1:500; Invitrogen, A-11007). Rat anti-HA (1:50; Cell Signaling, 11867423001, Clone 3F10) and Alexa Fluor 488-conjugated goat anti-rat secondary (1:500; Invitrogen, A-11006) antibodies were used to test for DMT1-3 × HA expression. Samples were all co-stained with SYTOX Deep Red (1 μM in dimethylsulfoxide (DMSO)) (Thermo Fisher) for nuclei, DyLight 405 NHS Ester (8 μM in DMSO) (Thermo Fisher) for RBC membrane/parasite plasma membrane/nuclear membrane, and also co-stained with MitoTracker Red CMXRos (50 nM; Invitrogen) or primary antibodies raised in mice or rabbits. Mouse anti-HA (1:250; Cell Signaling, 2367S, Clone 6E2) and Alexa Fluor 488-conjugated goat anti-mouse secondary (1:500; Invitrogen, A-21121) antibodies were used to probe for DMT1-3 × HA in co-stains with primary antibodies raised in rats.

For image acquisition, gels were cut and placed onto poly-D-lysine-coated 35 mm Cellvis imaging dishes (Fisher Scientific) and imaged with a Zeiss AxioObserver LSM 900 with Airyscan detector with ×63 magnification. All images were taken as *Z*-stacks with *Z*-slices at 0.15-μm intervals. Image analysis was done using ImageJ.

### DMT1 NF54 and Cam3.II protein expression and purification, and quinine and chloroquine transport measurements

The NF54 (quinine- and chloroquine-sensitive) and Cam3.II (quinine- and chloroquine-resistant) variants of the DMT1 and PfCRT proteins as well as control Band 3 protein (anion transport control) were expressed, purified and reconstituted in preformed liposomes as described previously^42^. The purified target proteins were reconstituted in preformed liposomes made of *E. coli* total lipids:cholesteryl hemisuccinate (CHS) 94:6 (w/w) following an established protocol^101,102^. The lumen of the proteoliposomes was composed of 100 mM KPi (pH 7.5) and 2 mM β-mercaptoethanol. Preformed liposomes were destabilized with 0.11% Triton X-100 and mixed with the target protein at a protein-to-lipid ratio of 1:150 (w/w). Detergent was removed by Bio-Bead SM-2 (Bio-Rad) treatment for 16 h at 4 °C. After separation of the suspension and Bio-Beads by filtration, the suspension was incubated at 4 °C for 30 min in the presence of 400 mM NaCl to remove non-incorporated protein from the proteoliposomes before ultracentrifugation of the samples (276,000 × *g* for 45 min at 4 °C). The preparations were washed in 100 mM KPi (pH 7.5) and 2 mM β-mercaptoethanol, subjected to ultracentrifugation, resuspended in the same buffer and shock frozen in liquid nitrogen until usage. Before measuring transport activity, the proteoliposomes were subjected to three freeze/thaw cycles and extrusion through 100-nm membrane filters (Avestin).

The full-length NF54 and Cam3.II *pfdmt1* were cloned into pEG BacMam vector^103^ with the C-terminal tobacco etch virus protease cleavage site (ENLYFQSYV) and a decahistidine affinity tag, followed by a streptavidin affinity tag (WSHPQFEK). For protein expression, the NF54 and Cam3.II bacmid and baculovirus were generated using the BacMam method^103^. The baculoviruses were produced in Sf9 cells (Expression System) and added to HEK293 GnTi^−^ cells (ATCC, CRL-3022) incubated at 37 °C for 20–24 h in the presence of 5% CO_2_ and 60% humidity. We then added 10 mM sodium butyrate (Sigma Aldrich) and further incubated the cells at 37 °C for 48 h before collecting them. Cell pellets were homogenized in low-salt buffer (10 mM HEPES pH 7.5, 10 mM KCl, 10 mM MgCl_2_, 0.5 mM PMSF, cOmplete EDTA-free protease inhibitor cocktail, 10 μg ml^−1^ DNase I and 8 μg ml^−1^ RNase) in a glass homogenizer. Membrane fractions were isolated by ultracentrifugation at 134,000 × *g* in a type 45 Ti rotor (Beckman Coulter). Membrane fractions were further homogenized and washed twice with high-salt buffer (10 mM HEPES pH 7.5, 10 mM KCl, 10 mM MgCl_2_, 1 M NaCl, 0.5 mM PMSF, cOmplete EDTA-free protease inhibitor cocktail, 10 μg ml^−1^ DNase I and 8 μg ml^−1^ RNase) in a glass homogenizer, followed by ultracentrifugation. The membrane fractions were homogenized and solubilized for 2 h in a buffer containing 20 mM HEPES pH 7.5, 200 mM NaCl, 0.5 mM PMSF, cOmplete EDTA-free protease inhibitor cocktail and 1% *n*-dodecyl-β-d-maltopyranoside DDM/0.1% CHS. Insoluble material was removed by ultracentrifugation at 134,000 × *g* for 30 min. The supernatant was added to pre-equilibrated Ni^2+^-NTA resin (Qiagen) in the presence of 20 mM imidazole and incubated at 4 °C overnight with gentle rotation. The protein-bound resin was washed with 10 column volumes of buffer containing 20 mM HEPES pH 7.5, 200 mM NaCl, 60 mM imidazole and 0.1% DDM/0.01% CHS. The protein was eluted in buffer consisting of 20 mM HEPES pH 7.5, 200 mM NaCl, 200 mM imidazole and 0.05% DDM/0.005% CHS. The eluted protein was further purified by loading on a Superdex 200 Increase 10/300 GL size-exclusion column (Cytiva) equilibrated with a buffer containing 20 mM HEPES pH 7.0, 150 mM NaCl and 0.025% DDM/0.0025% CHS. Purified proteins were reconstituted in preformed liposomes made of total *E. coli* lipids:CHS 97:3 (w/w) at a protein-to-lipid ratio of 1:150 (w/w). The lumen of the proteoliposomes was composed of 100 mM KPi pH 7.5 and 2 mM β-mercaptoethanol.

Uptake of [^3^H]chloroquine (300 nM, 1 Ci mmol^−1^) or [^3^H]quinine (300 nM, 1 Ci mmol^−1^) was measured after diluting PfCRT, DMT1 or Band 3-containing proteoliposomes in 50 μl of 100 mM Tris/MES (pH 5.5) in the presence or absence of the indicated compounds. Band 3 was used as a negative control, for which we expected no or minimal chloroquine and quinine uptake. Protein-specific uptake (pmol mg^−1^) was determined by subtracting the time-dependent accumulation of the tested compounds in control liposomes (lacking PfCRT, DMT1 or Band 3) from the accumulation measured in proteoliposomes containing one of these proteins. Data were collected in 3 independent experiments, performed in duplicate and are represented as the mean ± s.e.m. uptake at each timepoint.

### Inhibition of β-haematin formation assays

A solution containing deionized H_2_O/305.5 mM lipophilic detergent NP-40 Surfact-Amps Detergent solution (Thermo Fisher)/DMSO was prepared at a v/v ratio of 70%/20%/10%, respectively, and 100 μl was added to columns 1–11 of a flat-bottomed 96-well plate. Working stocks of test and control compounds were constituted to 20 mM (5 mM for md-amodiaquine), from which 20 μl (80 μl for md-amodiaquine) of each was added to wells in duplicate in column 12 together with deionized H_2_O (140 μl; 80 μl for md-amodiaquine) and 305.5 mM NP-40 detergent (40 μl). This effectively lowered the final drug concentration to 2 mM. Each compound (100 μl) was then serially diluted from columns 12 to 2 (column 1 served as a blank). A 25 mM haematin stock solution was prepared by sonicating haemin (Sigma) in DMSO for 3 min, and 178.8 μl of this solution was suspended in 20 ml of acetate buffer (1 M, pH 4.8) (made with sodium acetate (Sigma Aldrich) and acetic acid (Fisher Scientific)) and thoroughly mixed. The homogeneous suspension (100 μl) was then added to all wells to give a final 0.5 M buffer and 100 mM haematin concentration, as well as a 0.5 mM drug concentration in column 12. Plates were covered and incubated at 37 °C for 5 h, after which 32 μl 50% pyridine solution (20% (v/v) H_2_O, 20% (v/v) acetone, 10% (v/v) 2 M HEPES buffer (pH 7.4) and 50% (v/v) pyridine (Sigma Aldrich)) was added to each well. Acetone (60 μl) was then added to each well to assist with haematin dispersion. Unreacted haematin was quantified using the pyridine ferrihemochrome method^104^, which relies on aqueous pyridine specifically forming a low-spin complex with haematin but not β-haematin and the complex having an absorbance at 405 nm. The UV-vis absorbance of each well was read at 405 nm on a SpectraMax P340 plate reader. The β-haematin IC_50_ values for each compound were calculated from the absorbance values at 405 nm using sigmoidal dose–response curve fitting analysis (Prism v.10, GraphPad).

### LC–MS untargeted metabolomics

*Mycoplasma* contamination was assessed before sample collection using the e-Myco Mycoplasma detection PCR kit (BullDog-Bio). *Mycoplasma*-free parasites were sorbitol synchronized for at least two generations and magnetically enriched for 32 h post-invasion trophozoites using MACS CS columns (Miltenyi Biotec) to remove uninfected red blood cells. Trophozoite counts were determined by flow cytometery. Parasites (10^8^) were lysed in 1 ml 90% cold methanol containing 0.5 μM [^13^C_4_,^15^N_1_]-aspartate (Cambridge Isotope Laboratories) as an internal standard. Lysates were vortexed, centrifuged (13,000 × *g* for 10 min), and supernatants were collected. Any residual insoluble cell pellet was removed by centrifugation (20,000 × *g*, 4 °C for 20 min), and the 90% methanol supernatant was transferred to a new Eppendorf tube and dried under nitrogen gas flow. Samples were resuspended in HPLC-grade water containing 1 μM chlorpropamide (Alfa Aesar) as an internal standard. A quality-control (QC) sample was prepared by pooling equal volumes of each sample and assayed periodically to monitor LC–MS signal detection stability.

For LC–MS analysis, 5 μl of each sample was injected into a Shimadzu LC-40D XR HPLC/UHPLC pump. To separate metabolites, a reverse-phase method was used with a Waters BEH C18 column (100 mm × 2.1 mm, 1.7-μm particle size) set to 55 °C. Mobile phase A comprised HPLC-grade water with 0.1% formic acid and mobile phase B was HPLC-grade acetonitrile with 0.1% formic acid. The mobile phase gradient was as follows: 3% B at 0 min, 3% B at 0.01 min, 45% B at 10 min, 75% B at 12 min, 75% B at 17.5 min and 3% B from 18–20 min. The aqueous acetonitrile gradient ran for 20 min at a flow rate of 0.25 ml min^−1^. The resulting eluate was sent into a Sciex ZenoTOF 7600 system with a DuoSpray ion source. The spray voltage was 5.5 kV in positive mode and −4.5 kV in negative mode, while the declustering potential was 40 V in positive mode and −40 V in negative mode. Throughout the run, the curtain gas pressure was set to 35 psi, collision activated dissociation (CAD) gas to 9 psi, the ion source gas 1 and 2 both set to 50 psi and the temperature to 525 °C. Scanning range was from 50 to 1,000 *m*/*z* and 10 MS/MS product ion scans with a collision energy of 30 V with a 10 V spread.

Data analysis was performed as previously described^105^. Raw wiff2 data files were uploaded into MS-DIAL (v.5.1.231120)^106^ with the following parameters: MS1 tolerance 0.05 Da and MS2 tolerance 0.025 Da, minimum peak heat 1000 amplitude, mass slice width 0.1 Da, linear weighted moving average smoothing method with 3 scan level and minimum peak width 5 scans. Peak areas from negative and positive mode were input into a custom R script^107^ to annotate putative human haemoglobin (Hb) derived (either alpha or beta subunits, ≤13 amino acids) peptides (*m*/*z* matching within 15 ppm). The chlorpropamide peak was used to normalize metabolite peaks for instrument variability. Duplicate putative peptides were summed within the positive and negative mode peaks separately. If the same peak was called for multiple putative peptides, it was discarded. The relative standard deviation (RSD) was calculated from the QC samples for positive and negative peptides separately, and only those with an RSD < 30 were retained. Positive and negative mode duplicate peptides were combined, yielding 156 peptides in total. To assess the impact of mutations on the haemoglobin digestion of *P. falciparum*, the log_2_ fold changes in Cam3.II and select progeny relative to NF54 (WT) were calculated for each peptide from the average peak areas across all biological and technical replicates. A full list of peptides, detection mode and Hb subunit origin is provided in Supplementary Table 16.

### Inclusion and ethics statement

We gratefully acknowledge D. Ménard and colleagues who earlier collected the Cam3.II isolate from Cambodia and reported it in previous studies. This parasite and NF54, collected in the Netherlands from a *P. falciparum*-infected patient, were essential to the execution of this project. Red blood cells were purchased from the Interstate Blood Bank (Memphis, TN) as pooled, de-identified, anonymized blood that was washed to remove any residual leucocytes. Parasite culturing in these cells was approved by Institutional Ethics Committees at the Columbia University Irving Medical Center and the Johns Hopkins Bloomberg School of Public Health, who deemed this work to be Not Human Subjects Research under 45 CFR 46.

### Reporting summary

Further information on research design is available in the Nature Portfolio Reporting Summary linked to this article.

## Supplementary information

Supplementary Information Supplementary Figs. 1–9.

Reporting Summary

Supplementary Tables Supplementary Tables 1–20.

## Source data

Source Data Fig. 1 Drug response data.

Source Data Fig. 3 Drug response data, statistical source data.

Source Data Fig. 4 Drug response data, statistical source data.

Source Data Fig. 5 Drug uptake data, statistical source data.

Source Data Fig. 6 Drug response data, statistical source data.

Source Data Extended Data Fig. 1 β-haematin assay data.

Source Data Extended Data Fig. 3 Progeny and haplotype genome data.

Source Data Extended Data Fig. 5 Drug response data.

Source Data Extended Data Fig. 7 Clinical isolate genome data for *dmt1*, *ftsh1*, *samc* and *ftsh1*.

Source Data Extended Data Fig. 8 DMT1 co-localization data, statistical source data.

## Data Availability

All data needed to evaluate the conclusions in the paper are present in the paper and/or the supplementary materials. Raw reads of WGS data used in this study have been deposited in NCBI Sequenced Read Archive (SRA) under the BioProject accession number PRJNA1207213. LC–MS untargeted metabolomics data are available at the NIH Common Fund’s National Metabolomics Data Repository (NMDR) website, the Metabolomics Workbench (https://www.metabolomicsworkbench.org), with the Project ID ST004640. The data can be accessed directly via its Project at 10.21228/M8T27P (ref. ^108^). Source data are provided with this paper.

## Extended data

**Extended Data Table 1:** NF54 × Cam3.II genetic cross parents

Genetic Cross Parents	Geographical Origin (Year Adapted to Culture)						Resistance Phenotype (Geometric mean IC_50_ in nM)					
		*pfmdr1*		*pfcrt*	*k13*	*pm2/3*	CQ	md-CQ	QN	md-ADQ	MFQ	LMF
		Chr 5		Chr 7	Chr 13	Chr 14						
NF54	Africa (1979)	WT	1 copy	WT	WT	1 copy	S	S	S	S^hi^	S^hi^	S^hi^
							9.0	21.6	14.2	6.9	6.6	3.6
Cam3.II	Pursat, Cambodia (2010)	Y184F	2 copies	Dd2^a^	R539T	1 copy	R	R	R^mod^	S^lo^	S^lo^	S^lo^
							83.2	947.7	117.6	28.2	11.8	4.1

^a^Dd2 PfCRT haplotype: M74I/N75E/K76T/A220S/Q271E/N326S/I356T/R371I

*k13: kelch-13* (PF3D7_1343700); *pfcrt: chloroquine resistance transporter* (PF3D7_0709000); *pfmdr1: multidrug resistance protein 1* (PF3D7_0523000); *pm2/3: plasmepsins 2 and 3* (PF3D7_1408000 and PF3D7_1408100).

CQ: chloroquine; md-CQ: monodesethyl-chloroquine; QN: quinine; md-ADQ: monodesethyl-amodiaquine; MFQ: mefloquine; LMF: lumefantrine.

R, resistant; R^mod^, moderate resistance; S, sensitive; S^hi^, high sensitivity; S^lo^, low sensitivity; WT, wild-type.

Geometric mean IC_50_ values are listed in nM for parasites cultured in media with Albumax (no serum) (*N* = 4–85 with technical duplicates).

## Code availability

The custom R script used for analysis of the untargeted LC–MS metabolomics data is available in GitHub at https://github.com/syd-gav/Llinas-Lab-Codes (ref. ^107^).
