# Supplementary material for: Identification of genetic markers of quinine partial resistance in Plasmodium falciparum
Source: Nat Microbiol. 2026 Jul 6;11(8):2213–31. doi: 10.1038/s41564-026-02410-7 (PMC13423875; doi:10.1038/s41564-026-02410-7)
Supplement: Supplementary file 1 — Supplementary Figs. 1–9. [file 41564_2026_2410_MOESM1_ESM.pdf]

nature microbiology

Supplementary information https://doi.org/10.1038/s41564-026-02410-7

Identification of genetic markers of quinine partial resistance in *Plasmodium falciparum*

In the format provided by the authors and unedited

Kanai *et al.* Identification of genetic markers of quinine partial resistance in *Plasmodium falciparum*

# – Supplementary Figures

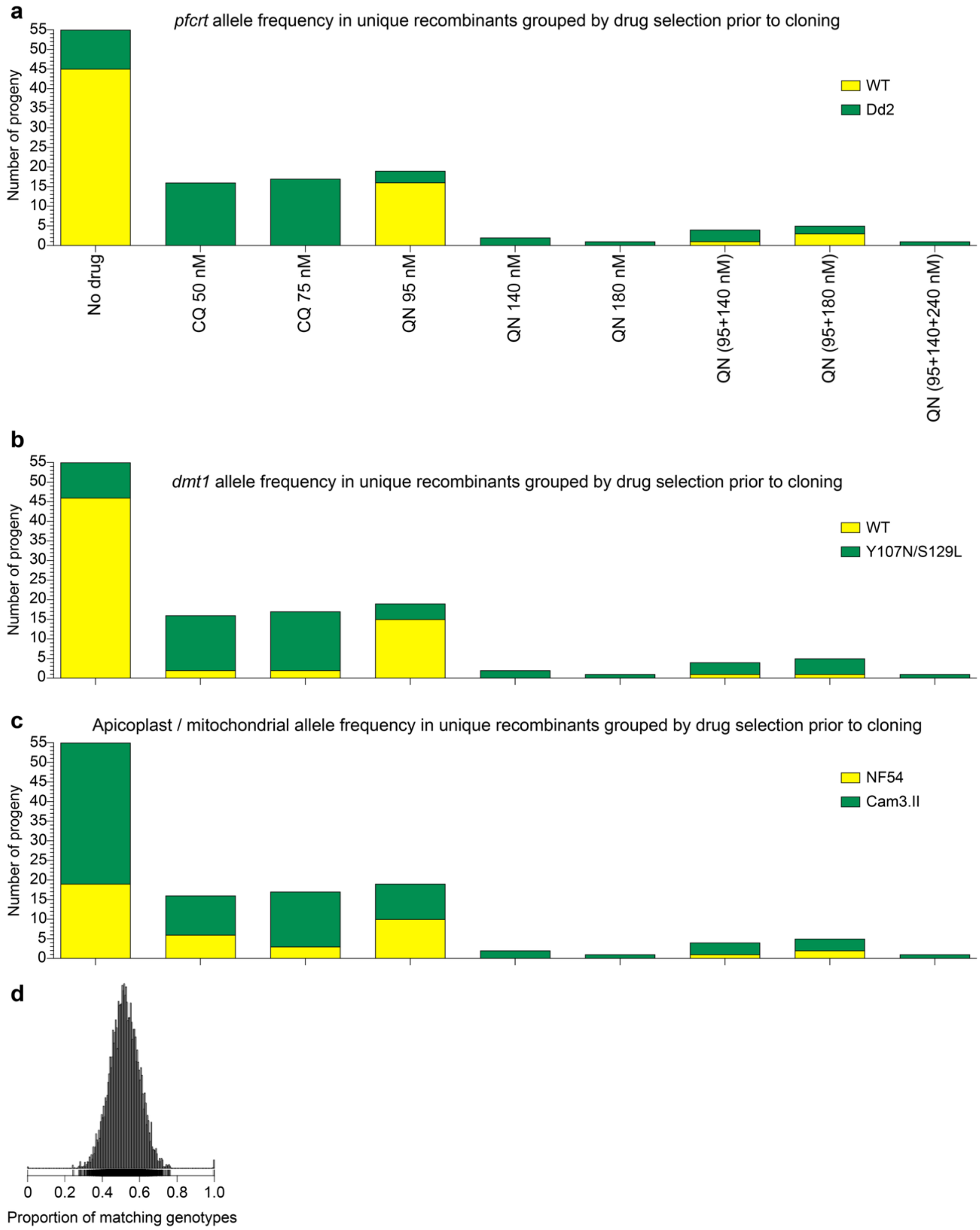

Supplementary Fig. 1 | *pfcrt*, *dmt1*, and apicoplast/mitochondrial allele frequencies in the recombinant progeny. a-c, Number of unique recombinant progeny with the wild-type NF54 or

mutant Cam3.II alleles for (**a**) *pfcrt,* (**b**) *dmt1*, and (**c**) the apicoplast and mitochondrial genomes. Data are grouped by the selection condition applied before obtaining the progeny clone. If a unique recombinant haplotype was obtained in more than one selection pressure condition, this was shown in every corresponding group. CQ, chloroquine; QN, quinine. The apicoplast and mitochondrial haplotypes are: Cam3.II: *PF3D7_API00100*, S25G; *PF3D7_API02100*, V46I; *PF3D7_API02300*, K118R; *PF3D7_API03600*, L195; *PF3D7_API04400*, S658; *mal_mito_1*, wild-type; NF54: wild-type for all apicoplast genes; *mal_mito_1*, I250V. **d**, Genetic relatedness between the 120 recombinant progeny clones and two parents was measured by identity-by-descent in multiple pairwise comparisons.

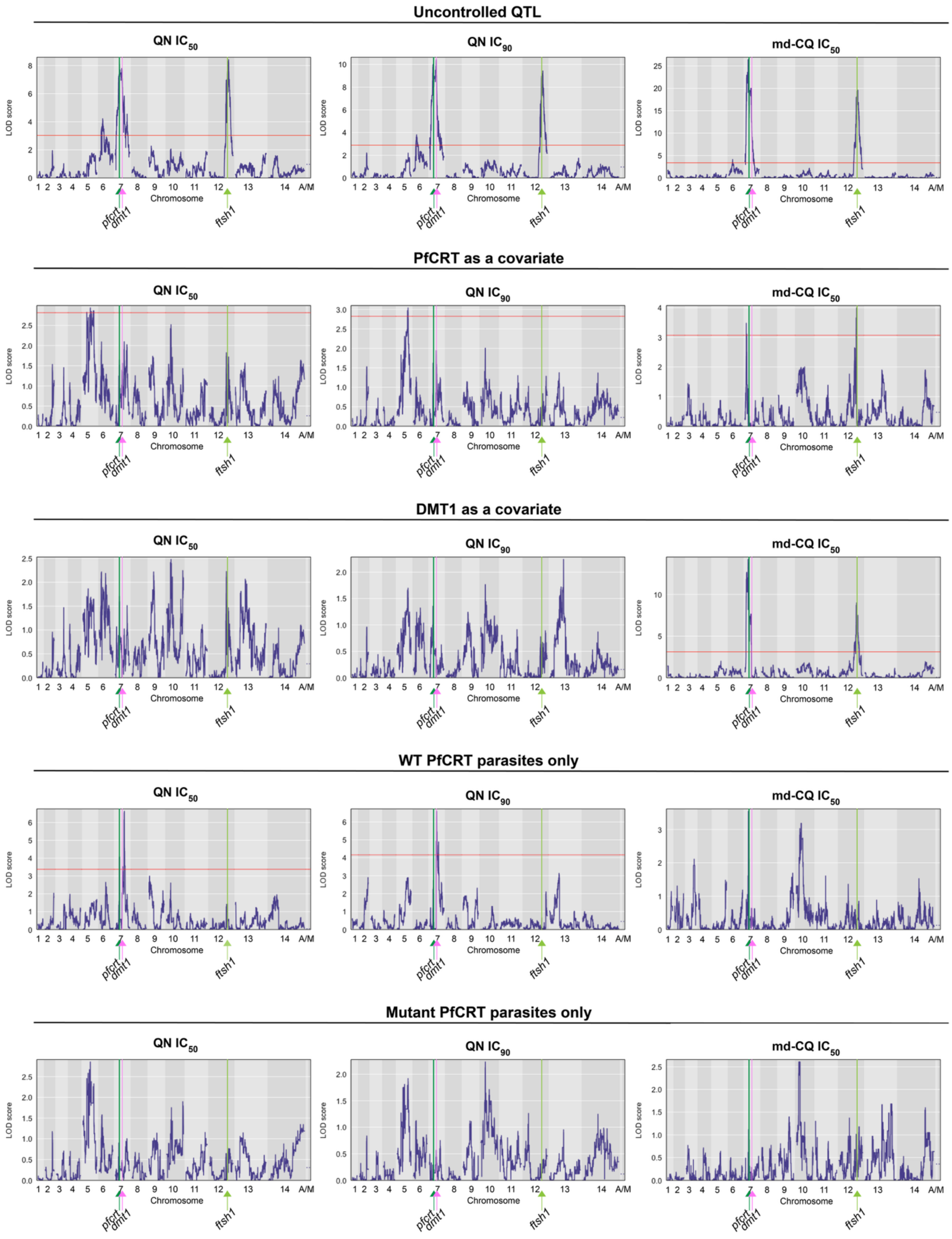

Supplementary Fig. 2 | Further QTL mapping of QN and md-CQ response with *pfcrt* or *dmt1* as a covariate, or by stratifying by *pfcrt* allele. LOD plots for geometric mean QN IC_{50}. QN

IC_{90}, and md-CQ IC_{50} values using data associated with the cross parents and 90, 85, and 80 progeny, respectively. The red line indicates the 95% probability threshold. QTL, quantitative trait locus; LOD, logarithm of the odds; md-CQ, monodesethyl-chloroquine; QN, quinine; IC_{50} and IC_{90}, 50% and 90% growth inhibitory concentrations. In the top row, data from **Fig. 2a-c** are shown again for visual comparison.

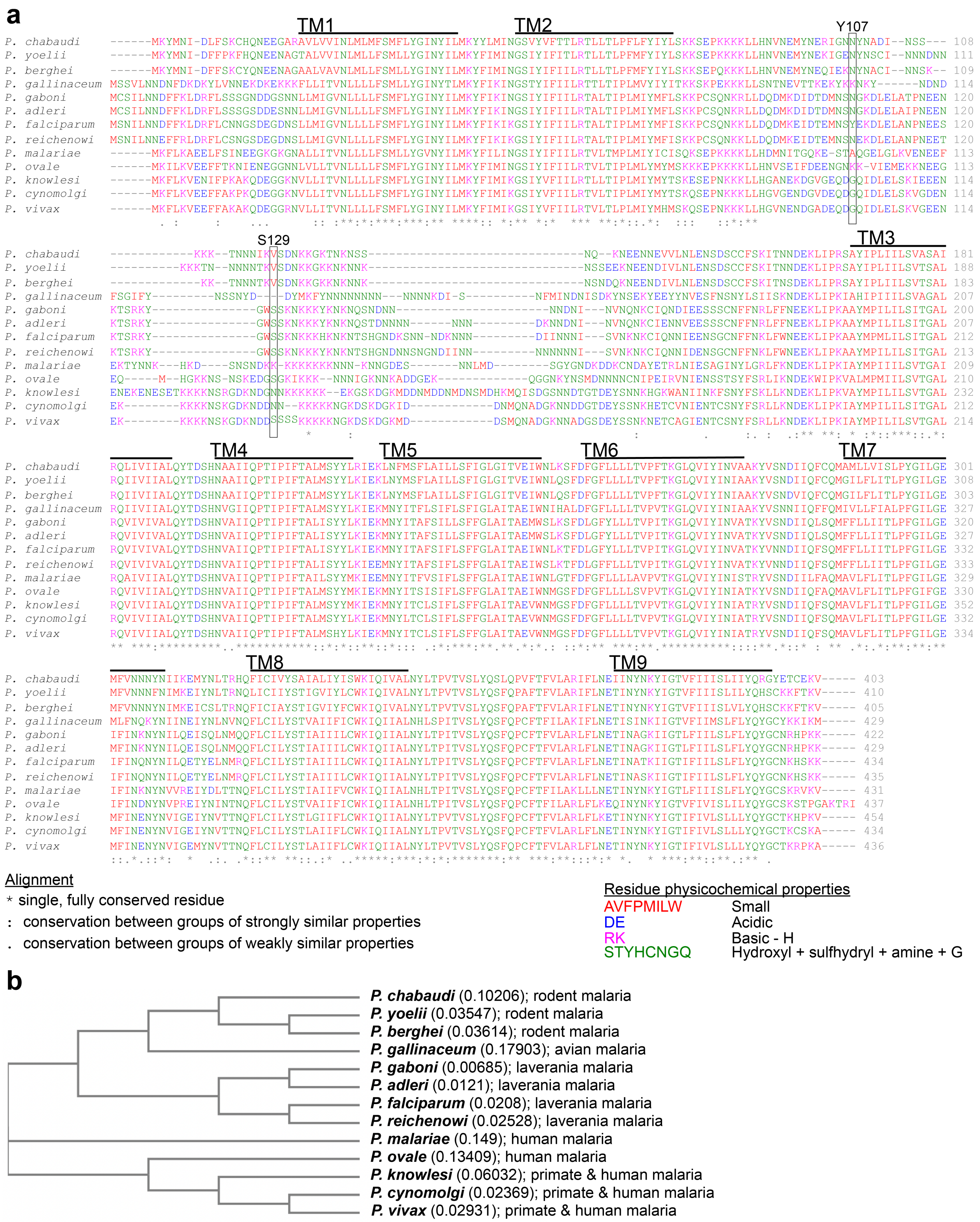

Supplementary Fig. 3 | Residues in predicted transmembrane regions of PfDMT1 show conservation between orthologues of DMT1 in other *Plasmodium spp.* a, Multiple sequence alignment of NF54 *P. falciparum* DMT1 with sequences of twelve orthologs of DMT1 sequences

in rodent (*P. chabaudi*: chabaudi, *P. yoelii*: 17X, *P. berghei*: ANKA), avian (*P. gallinaceum*: 8A), laverania (*P. gaboni*: SY75*, P. adleri*: G01, *P. reichenowi*: CDC), human (*P. malariae*: UG01, *P. ovale*: GH01, *P. knowlesi*: H), and primate / human (*P. cynomolgi*: M, *P. vivax*: P01) reference *Plasmodium* species. The residues corresponding to the Y107N and S129L mutations in *P. falciparum* Cam3.II, and the TMHMM-predicted transmembrane domains of PfDMT1 (dark line) are indicated. **b**, Phylogenetic tree of the DMT1 sequences from the 13 *Plasmodium spp.* based on their multiple sequence alignment, with the tree “lengths” shown in parentheses (indicative of the evolutionary distance and genetic change between sequences). *P. falciparum* DMT1 was the most similar to laverania orthologues, which all carried Y107N and WT S129.

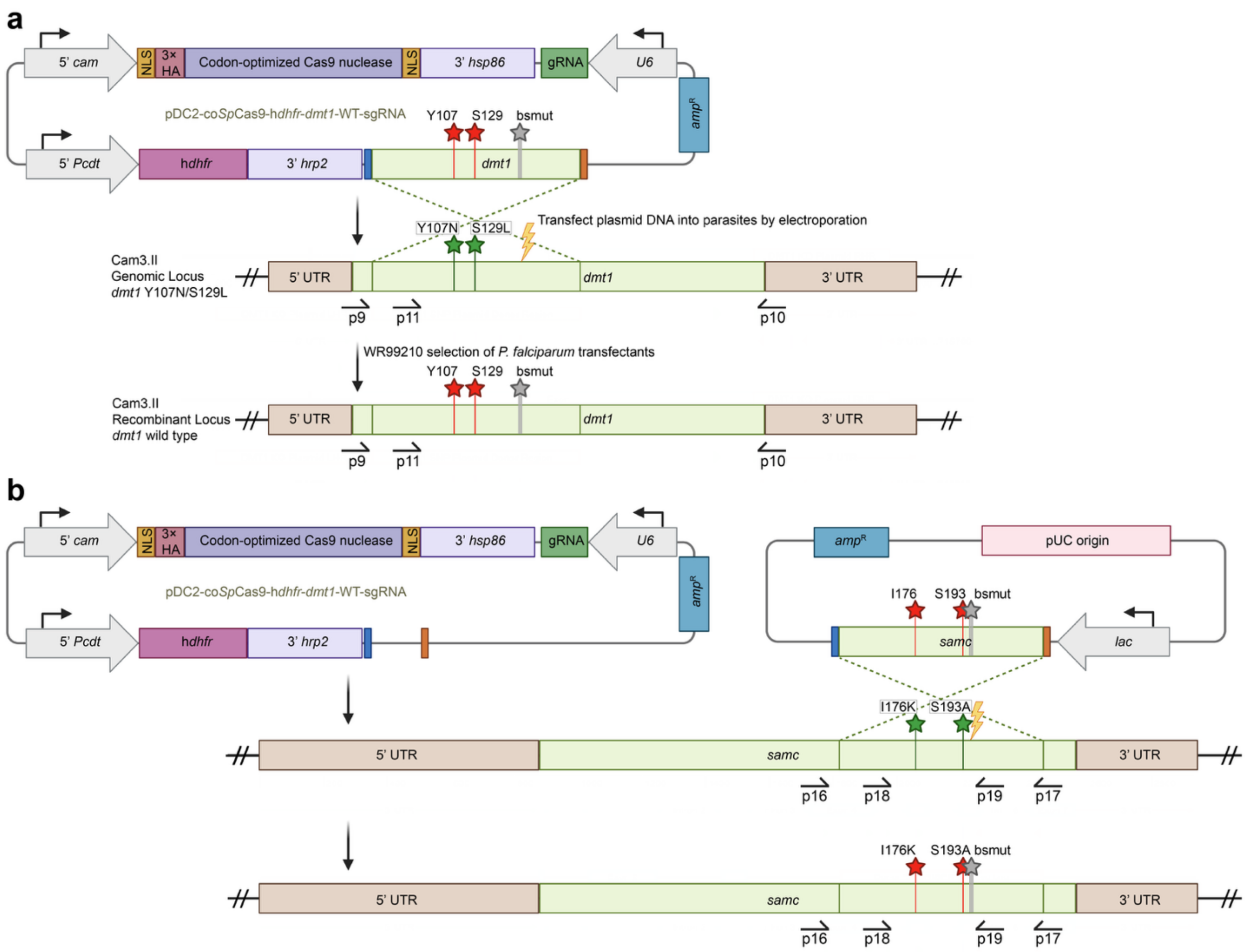

Supplementary Fig. 4 | CRISPR/Cas9 plasmid construction and editing strategy for single nucleotide polymorphism (SNP) editing*.* **a**, All-in-one plasmid approaches used for CRISPR/Cas9-mediated SNP editing of QTL candidate genes in the cross parents and progeny, with the *dmt1* gene shown as a representative. This plasmid contained the *cam* promoter to express codon-optimized Cas9 and a *PcDT* promoter to express the WR99210-selectable h*dhfr* marker. This schematic shows a door fragment encoding the two *dmt1* wild-type codons Y107 and S129 along with binding-site mutations (bsmut; shown as grey stars) to prevent further cleavage of gene-edited parasites. Plasmid DNA was transfected into parasites by electroporation to initiate a double-stranded break, and subsequent homology-directed repair and replacement of the mutated codons encoding Y107N and S129L (green stars) with the wild-type sequence (red stars). Donor plasmids were also designed with mutant codons that were gene edited into parasites with wild-type *dmt1*. **b**, The two-plasmid strategy used for CRISPR/Cas9-mediated SNP editing of *samc*. The donor for SAMC was kept in the pUC-GW-*amp* vector and co-transfected with a Cas9 plasmid expressing the gRNA. *cam*, calmodulin; gRNA, guide RNA; h*dhfr*, human dihydrofolate reductase; *hrp2*: histidine-rich protein 2; *hsp86*, heat shock protein 86; *PcDT*, *P. chabaudi* dihydrofolate reductase-thymidylate synthase; *amp*^{R}, ampicillin resistance gene; *dmt1*, *P. falciparum* drug/metabolite transporter 1; UTR, untranslated region. Primers used for cloning and verification are described in **Supplementary Table 10**. Plasmids are described in **Supplementary Table 11**.

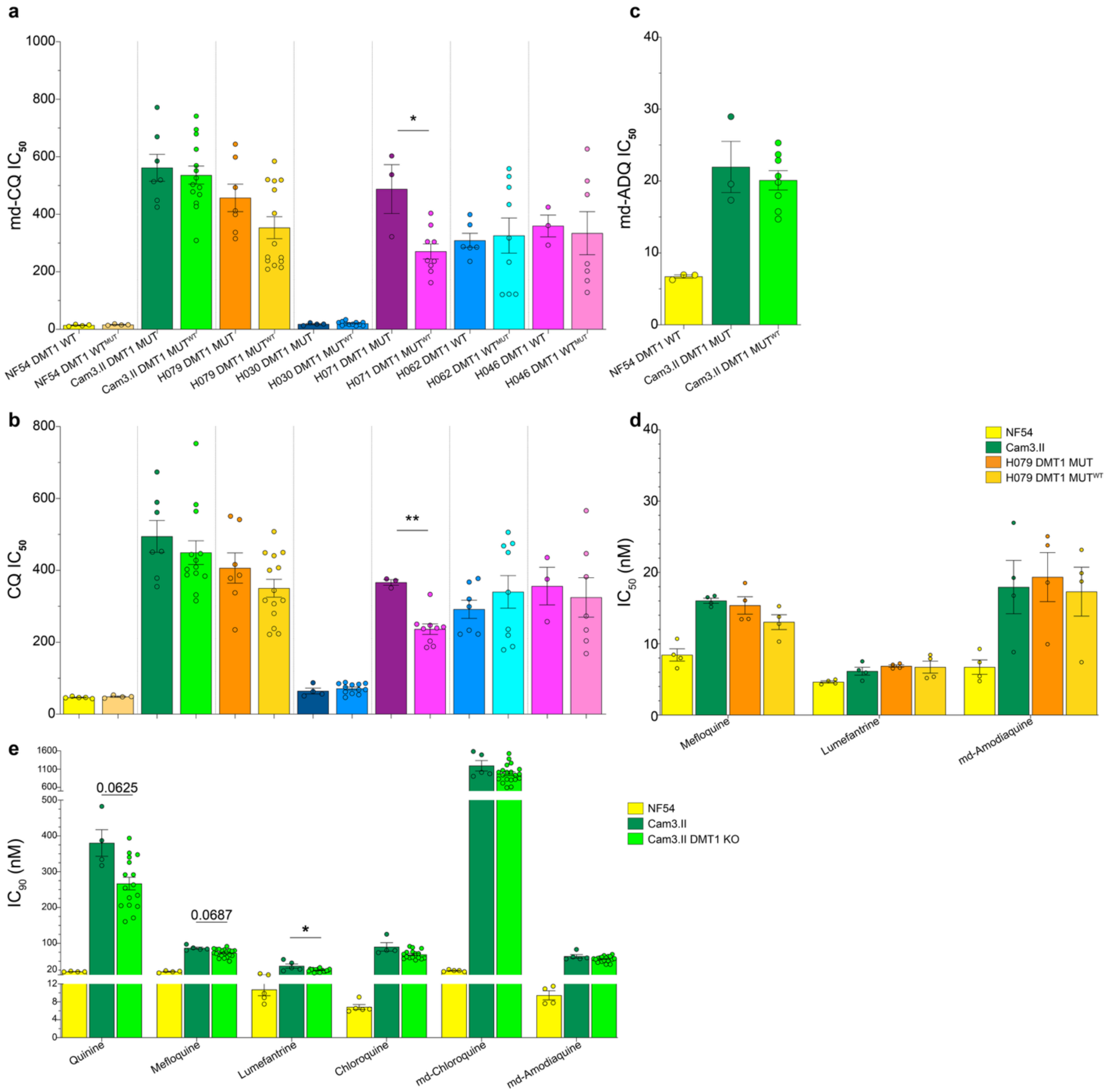

Supplementary Fig. 5 | Phenotypic responses of *dmt1* gene-edited parents and progeny to monodesethyl-chloroquine (md-CQ), chloroquine (CQ), and monodesethyl-amodiaquine (md-ADQ). a-c, md-CQ (**a**), CQ (**b**), and md-ADQ (**c**) mean ± SEM IC_{50} values. For each parasite background, we phenotyped a parental unedited parasite (endogenous DMT1 haplotype) (dark color) and its isogenic gene-edited line (with binding-site mutations and either a Y107N / S129L mutant or a wild-type revertant) (light bright color). *p*-values (unpaired, two-sided Student's *t*-test, **a-c** corrected with the Bonferroni-Šídák method) are indicated (N=3–14 biological replicates, with technical duplicates). **d**, Mefloquine, lumefantrine, and md-ADQ response in the H079 progeny DMT1 revertant as measured by mean ± SEM IC_{50} values. Results shown in panels **a-c** revealed no statistically significant differences, except for the H071 isogenic pair. H071 DMT1 revertants became less resistant to CQ and md-CQ than their unedited, CQ-resistant isogenic line, possibly due to an altered interaction with another gene product in this recombinant background. The Cam3.II DMT1 revertant parasites did not become less resistant to md-CQ, CQ, and md-ADQ,

compared with their isogenic DMT1 mutant lines. For H079, for which the DMT1 revertant (*dmt1*^{WT}) was significantly sensitized to QN, the isogenic pair was also profiled against MFQ, LMF, and md-ADQ (**d**). Those assays found no statistically significant difference. **e**, Quinine, mefloquine, lumefantrine, CQ, md-CQ, and md-ADQ response in Cam3.II DMT1 knockout parasites as measured by mean ± SEM IC_{90} values (N=4 biological replicates, with technical duplicates). *p*-values (unpaired, two-sided Student's *t*-test, corrected with the Bonferroni-Šídák method) are indicated for the Cam3.II KO strain vs the Cam3.II parent (N=4–21 biological replicates, with technical duplicates). *$p < 0.05$, **$p < 0.01$, ***$p < 0.001$. WT: wild-type; MUT: Y107N / S129L mutation. Values are listed in **Supplementary Table 12**.

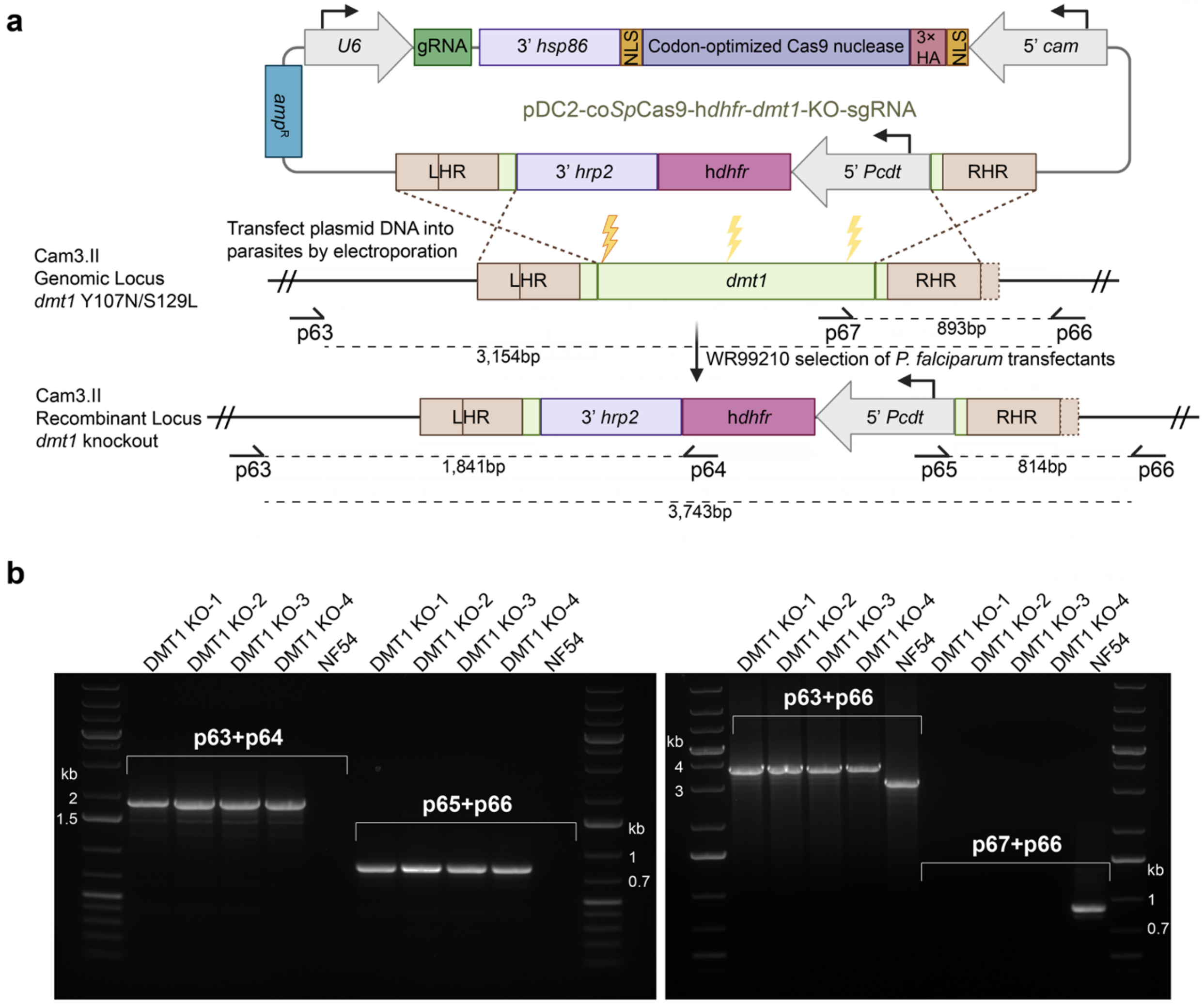

Supplementary Fig. 6 | CRISPR/Cas9-based generation of DMT1 knockout Cam3.II parasites.

The all-in-one CRISPR/Cas9-based DMT1 knockout tagging strategy. **a**, CRISPR/Cas9 plasmids included a *dmt1* left homology region (LHR) and right homology region (RHR) flanking a human dhfr selectable marker cassette. Expression of Cas9 and a gRNA led to a double-stranded break in *dmt1*, which was repaired using the LHR and RHR homologous regions. This resulted in introduction of the selectable marker cassette (selected using 2.5 nM WR99210) and a dmt1 knockout (KO). Three plasmids were generated, each with its own gRNA (yellow thunderbolts) that targeted an internal *dmt1* sequence that was deleted during the gene editing event. **b**, PCR and gel electrophoresis-based verification of complete *dmt1* knockout in three Cam3.II parasite replicates, compared to a NF54 unedited control. Results confirmed the presence of the 5'-incorporated h*dhfr* cassette (p63+p64), 3'-incorporated *PcDT* promoter (p65+p66), ~600 bp larger size of the DMT1 recombinant knockout locus (p63+p66), and lack of the deleted *dmt1* locus (p67+p66). PCR primers and expected PCR product sizes are indicated above in (**a**). Primers used for verification and cloning are described in **Supplementary Table 10**. Plasmids are described in **Supplementary Table 11**.

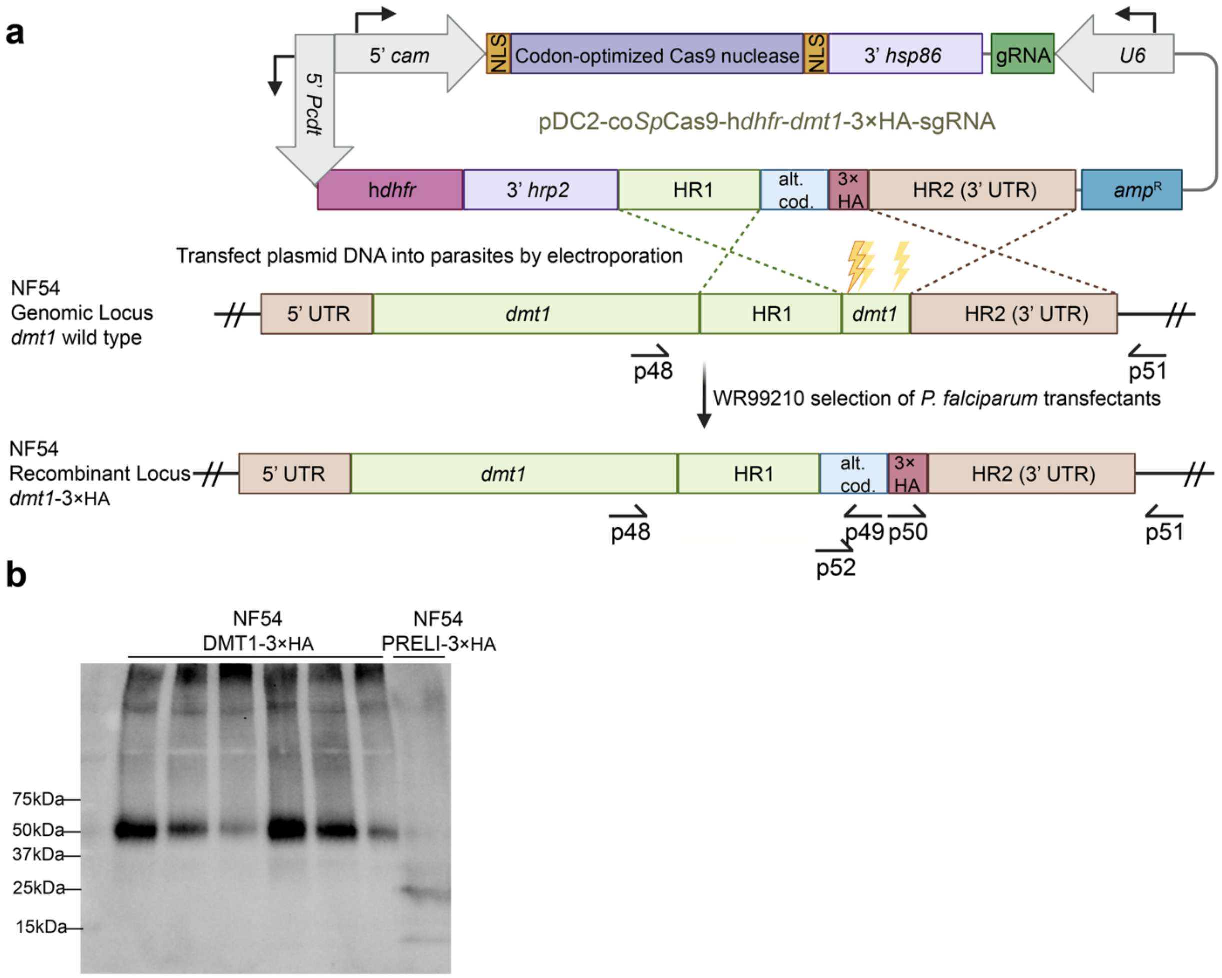

Supplementary Fig. 7 | CRISPR/Cas9-based generation of DMT1 3' 3×HA-tagged NF54 parasites. a, The all-in-one CRISPR/Cas9-based DMT1 3' 3×HA tagging strategy. CRISPR/Cas9 plasmids were constructed as depicted in **Supplementary Fig. 4**, except the donor template included a homology region 1 (HR1) (342 bp), a recodonized 3' end of *dmt1* to disrupt homology with no stop codon (165 bp), a filler sequence (6 bp), a 3×HA epitope tag followed by a stop codon, and a 3' untranslated region homology region 2 (HR2; 509 bp). Three plasmids were generated, each with a separate gRNA located within the recodonized region. Thus, binding-site mutations were not necessary. **b**, Western blot of NF54 DMT1-3×HA (showing an expected 50 kDa band) and NF54 PRELI-3×HA parasites as a 3×HA positive control (~25 kDa band expected). Parasite lysates were incubated with an anti-HA antibody. The Western blot experiment was conducted twice. Primers used for cloning and verification are described in **Supplementary Table 10**. Plasmids are described in **Supplementary Table 11**.

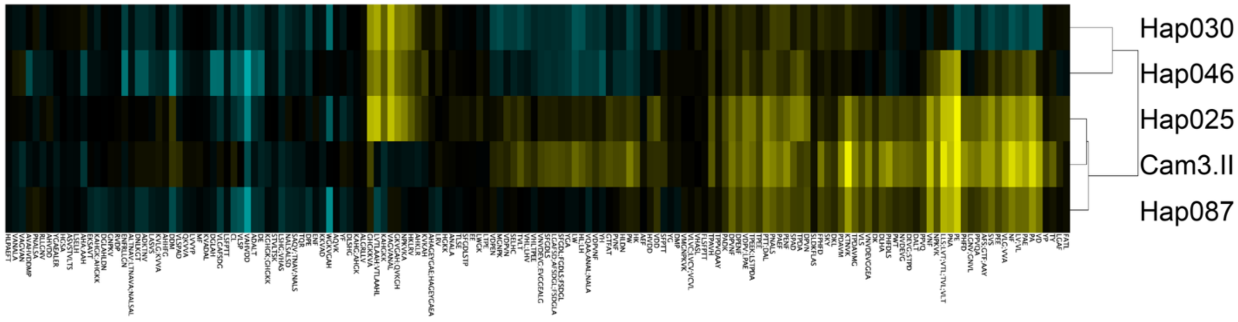

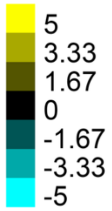

Haplotype Group	Phenotyped Parasite	*pfcrt (chr 7)*	*pfdmt1 (chr 7)*	*pfthzk (chr 12)*	*pfftsh1 (chr 12)*	*pfsamc (chr 12)*
x2_Hap001_NF54	x2_NF54_JHU	WT	WT	WT	WT	WT
x2_Hap030	x2_B4-5	WT	Y107N/S129L	N244/A301V	D695G	I176K/S193A
x2_Hap046	x2_B5-4	Dd2	WT	N244/A301V	D695G	I176K/S193A
x2_Hap025	x2_B7-5	Dd2	WT	WT	WT	WT
x2_Hap002_Cam3.II	x2_Cam3.II_G8	Dd2	Y107N/S129L	N244/A301V	D695G	I176K/S193A
x2_Hap087	x2_C-CQ75-1-G12	Dd2	Y107N/S129L	WT	WT	WT

Supplementary Fig. 8 | Heatmap of 156 putative hemoglobin-derived peptides levels in cross parents and select progeny. Differences in peptide accumulation (yellow) or depletion (blue) were computed as $\log_2$ fold changes of the average abundance for each peptide from each parasite line identified compared to NF54 (N=3 biological replicates, with technical triplicates). Parasite lines (x-axis) and peptides (y-axis) were subjected to hierarchical average linkage clustering. Raw counts and peptide listings are included in **Supplementary Table 16**.

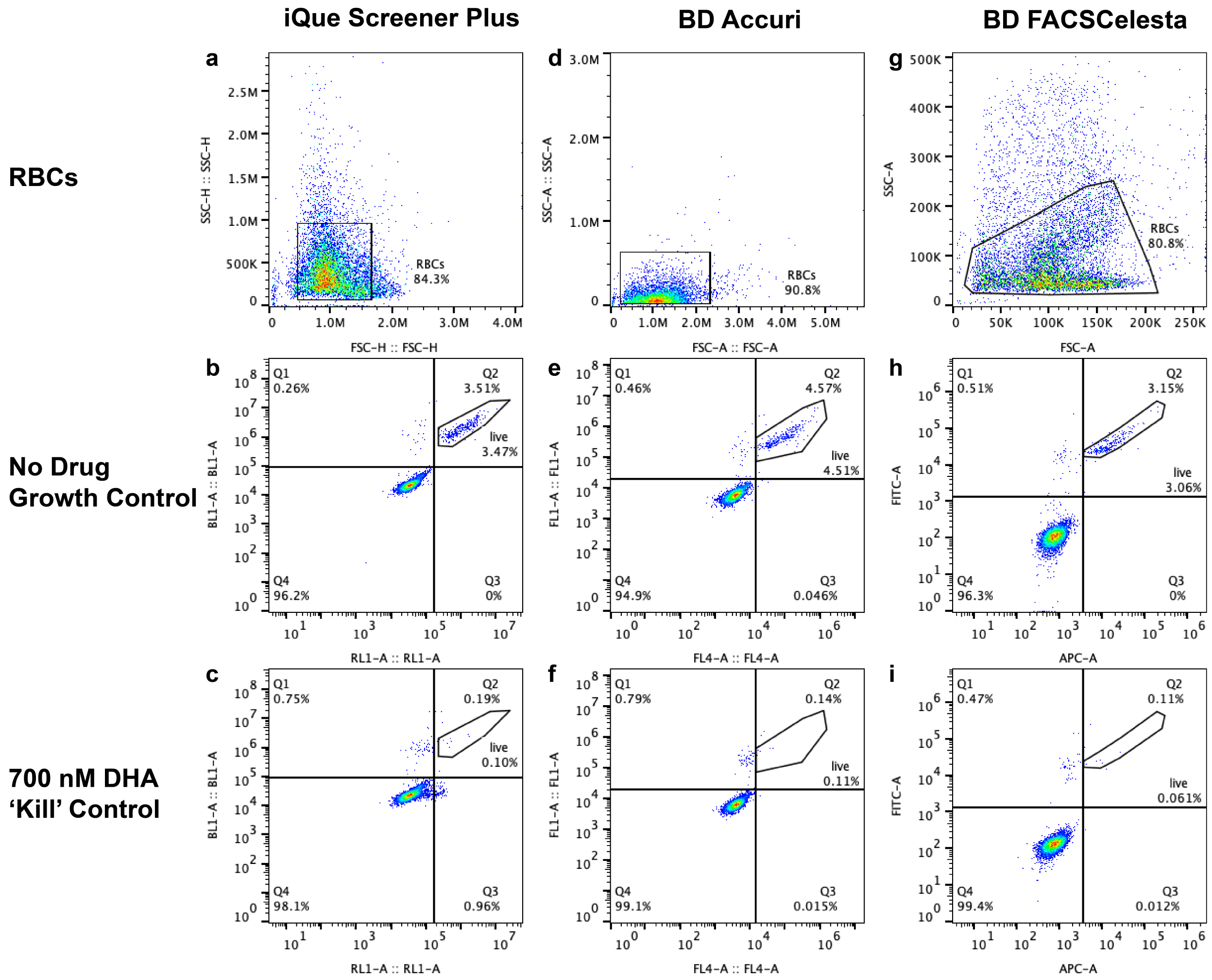

Supplementary Fig. 9 | Flow cytometry gating strategy to quantify parasitemias in drug-susceptibility assays. Cells were stained with 1×SYBR Green I and 100 nM MitoTracker DeepRed FM for at least 20 min and diluted in 1×PBS prior to sampling (typically ~8,000-10,000 RBCs per well). Parasitemias were measured using an iQue Screener Plus (**a-c**), BD Accuri C6 (**d-f**), or BD FACSCelesta flow cytometer (**g-i**). Cells were first gated by forward (FSC) and side (SSC) scatter channels to exclude extracellular debris and to select for red blood cells (**a,d,g**). Live parasites in each well were determined as the percentage of MitoTracker-positive SYBR Green I-positive infected RBCs within the RBC subpopulation gated in the FSC vs SSC plot (upper right gate). Thus, live parasites were identified as BL1-A$^+$/RL1-A$^+$ on the iQue (**b,c**), FL1-A$^+$/FL4-A$^+$ on the Accuri (**e,f**), and FITC-A$^+$/APC-A$^+$ on the FACSCelesta (**h,i**). For each parasite line, no drug controls were used to determine full parasite growth (**b,e,h**) and 700 nM dihydroartemisinin (DHA) was used as a 'kill' control to determine background (**c,f,i**). Parasitemia was then normalized by subtracting the background parasitemia in the 700 nM DHA kill control, and IC$_{50}$ and IC$_{90}$ values were calculated by nonlinear regression analysis.

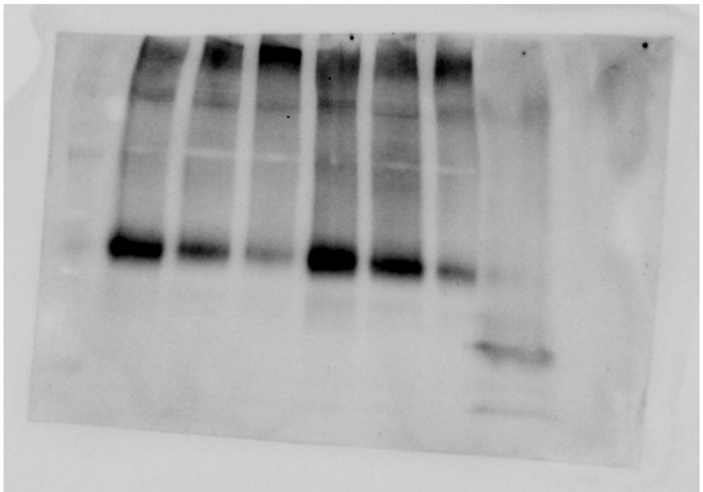

Uncropped Western blot of NF54 DMT1-3×HA (showing an expected 50 kDa band) and NF54 PRELI-3×HA parasites as a 3×HA positive control (~25 kDa band expected) shown in **Supplementary Figure 7b**. Parasite lysates were incubated with an anti-HA antibody.
