# Supplementary material for: Identification of genetic markers of quinine partial resistance in Plasmodium falciparum
Source: Nat Microbiol. 2026 Jul 6;11(8):2213–31. doi: 10.1038/s41564-026-02410-7 (PMC13423875; doi:10.1038/s41564-026-02410-7)
Supplement: Supplementary file 2 — Reporting Summary [file 41564_2026_2410_MOESM2_ESM.pdf]

nature portfolio

Corresponding author(s): David A. Fidock, Ph.D.

Last updated by author(s): Jun 12, 2026

Reporting Summary

Nature Portfolio wishes to improve the reproducibility of the work that we publish. This form provides structure for consistency and transparency in reporting. For further information on Nature Portfolio policies, see our Editorial Policies and the Editorial Policy Checklist.

Statistics

For all statistical analyses, confirm that the following items are present in the figure legend, table legend, main text, or Methods section.

n/a	Confirmed	
☐	☒	The exact sample size (*n*) for each experimental group/condition, given as a discrete number and unit of measurement
☐	☒	A statement on whether measurements were taken from distinct samples or whether the same sample was measured repeatedly
☐	☒	The statistical test(s) used AND whether they are one- or two-sided<br>*Only common tests should be described solely by name; describe more complex techniques in the Methods section.*
☐	☒	A description of all covariates tested
☐	☒	A description of any assumptions or corrections, such as tests of normality and adjustment for multiple comparisons
☐	☒	A full description of the statistical parameters including central tendency (e.g. means) or other basic estimates (e.g. regression coefficient) AND variation (e.g. standard deviation) or associated estimates of uncertainty (e.g. confidence intervals)
☐	☒	For null hypothesis testing, the test statistic (e.g. *F*, *t*, *r*) with confidence intervals, effect sizes, degrees of freedom and *P* value noted<br>*Give P values as exact values whenever suitable.*
☒	☐	For Bayesian analysis, information on the choice of priors and Markov chain Monte Carlo settings
☒	☐	For hierarchical and complex designs, identification of the appropriate level for tests and full reporting of outcomes
☒	☐	Estimates of effect sizes (e.g. Cohen's *d*, Pearson's *r*), indicating how they were calculated

Our web collection on statistics for biologists contains articles on many of the points above.

Software and code

Policy information about availability of computer code

Data collection	Drug inhibition assays: FlowJo Version 10.10.0 (FlowJo, LLC)<br>Gene editing strategy and validation: SeqBuilder Pro version 17.4.1 (DNASTAR), SeqMan Ultra version 17.4.1 (DNASTAR), Benchling<br>gRNA selection: ChopChop version 3 (University of Bergen) and Benchling<br>Immunofluorescence assays: NIS-Elements version 5.02 (Nikon)
Data analysis	Drug inhibition assays, [3H]-CQ and [3H]-QN transport assays, Beta-hematin inhibition assays: Prism version 10.0 (GraphPad)<br>Progeny-clone based QTL analysis: r/QTL version 2<br>Bulk segregant analysis: r/QTLseqr<br>Genetic map construction: JoinMap 5 (Kyazma)<br>Genome analysis: PlasmoDB (version 48), Burrow-Wheeler Alignment, SAMtools, Picard, Genome Analyses Tool Kit (GATK) RealignerTargetCreator, GATK BaseRecalibrator, SnpEff<br>Haplotype genome visualization and haplotype assignment: Gene Cluster 3.0, Java TreeView<br>Microsatellite size identification: a custom Python script utilizing the pysam module (previously published in citation 80)<br>Immunofluorescence assays: Imaris version 9 (Oxford Instruments), Fiji version 2.11.0 (ImageJ)<br>Sequence alignment: Clustal Omega (EMBL-EBI), EMBOSS Needle (EMBL-EBI)<br>Ultrastructure expansion microscopy: Fiji version 2.11.0 (ImageJ)<br>LC-MS untargeted metabolomics: MS-DIAL version 5.1.231120 and custom R script deposited at https://github.com/syd-gav/Llinas-Lab-Codes

For manuscripts utilizing custom algorithms or software that are central to the research but not yet described in published literature, software must be made available to editors and reviewers. We strongly encourage code deposition in a community repository (e.g. GitHub). See the Nature Portfolio guidelines for submitting code & software for further information.

Data

Policy information about availability of data

All manuscripts must include a data availability statement. This statement should provide the following information, where applicable:

- Accession codes, unique identifiers, or web links for publicly available datasets
- A description of any restrictions on data availability
- For clinical datasets or third party data, please ensure that the statement adheres to our policy

All data needed to evaluate the conclusions in the paper are present in the paper and/or the Supplementary Materials. Raw reads of WGS data used in this study have been deposited in NCBI Sequenced Read Archive (SRA) under the BioProject accession number PRJNA1207213. LC/MS untargeted metabolomics data are available at the NIH Common Fund's National Metabolomics Data Repository (NMDR) website, the Metabolomics Workbench, https://www.metabolomicsworkbench.org, with the Project ID ST004640. The data can be accessed directly via it's Project DOI: 10.21228/M8T27P.

Research involving human participants, their data, or biological material

Policy information about studies with human participants or human data. See also policy information about sex, gender (identity/presentation), and sexual orientation and race, ethnicity and racism.

Reporting on sex and gender	Columbia University: Human red blood cells were purchased from a commercial blood bank and were pooled from anonymous blood donors. There are no identifiers that would enable the donors to be identified. Therefore no data on sex and gender were collected.<br>Johns Hopkins University: Human red blood cells were obtained weekly from healthy male volunteers. There are no data that the parasite prefers erythrocytes from male versus female volunteers.
Reporting on race, ethnicity, or other socially relevant groupings	Columbia University: Human red blood cells were purchased from a commercial blood bank and were pooled from anonymous blood donors. There are no identifiers that would enable the donors to be identified. Therefore no data on race, ethnicity, or other socially relevant groupings were collected.<br>Johns Hopkins University: Human red blood cells were obtained weekly from healthy male volunteers of any race or ethnicity.
Population characteristics	Columbia University and Johns Hopkins University: No data on population characteristics were collected.
Recruitment	Johns Hopkins University: Human red blood cells used to set up the cultures were collected weekly from healthy volunteers.<br>Columbia University: Human red blood cells were purchased from a commercial blood bank and were pooled from anonymous blood donors. There are no identifiers that would enable the donors to be identified. Therefore there was no participant recruitment.
Ethics oversight	Johns Hopkins University: Human red blood cells used to set up the cultures were collected weekly from healthy volunteers, with informed consent, under a Johns Hopkins University Institutional Review Board approved protocol (number NA_00019050). All experiments were performed in accordance with institutional guidelines and regulations.<br>Columbia University: A research protocol (IRB-AAAC4249) was submitted by Dr. Fidock to the Institutional Review Board (IRB) at the Columbia University Irving Medical Center (CUIMC). This protocol was approved on 22 September 2022 by the IRB as "not human subjects research in accordance with the Code of Federal Regulations Title 45 - Public Welfare Department of Health and Human Services, Part 46 - Protection of Human Subjects."

Note that full information on the approval of the study protocol must also be provided in the manuscript.

Field-specific reporting

Please select the one below that is the best fit for your research. If you are not sure, read the appropriate sections before making your selection.

☒ Life sciences ☐ Behavioural & social sciences ☐ Ecological, evolutionary & environmental sciences

For a reference copy of the document with all sections, see nature.com/documents/nr-reporting-summary-flat.pdf

Life sciences study design

All studies must disclose on these points even when the disclosure is negative.

Sample size	Our study provided paired phenotypic and whole-genome sequence data for 50-90 progeny (depending on the drug profiled) and the parents, which was deemed sufficient in light of earlier genetic cross work that successfully determined resistance traits using ~35 progeny.
Data exclusions	Dose-response assays were excluded if the maximum parasitemia in the absence of drug was below 1% or greater than 8%. We also excluded assays if the IC90/IC50 ratio was substantially skewed relative to other comparative studies with a given parasite line and drug combination.
Replication	Dose-response assays almost always were performed on at least five independent occasions, with technical duplicates. Parental lines were systematically included as benchmarks in each round of assays.
Randomization	For the bulk segregant analysis, bulk progeny pools were randomly assigned to quinine pressure or control groups by creating aliquots for each pressure condition. Random allocation of samples into experimental groups was not applicable for individual progeny-based drug assays,

	as we primarily assayed one progeny per haplotype, which was assigned based on its genotype, and we assayed CRISPR/Cas9-edited isogenic parasite lines for biological differences.
Blinding	Not applicable as QTL analyses inherently requires linking each progeny's genotype and phenotype, and the underlying whole-genome sequences are objective and the drug susceptibility data are quantitative.

Reporting for specific materials, systems and methods

We require information from authors about some types of materials, experimental systems and methods used in many studies. Here, indicate whether each material, system or method listed is relevant to your study. If you are not sure if a list item applies to your research, read the appropriate section before selecting a response.

Materials & experimental systems

n/a	Involved in the study
☐	☒ Antibodies
☐	☒ Eukaryotic cell lines
☒	☐ Palaeontology and archaeology
☐	☒ Animals and other organisms
☒	☐ Clinical data
☒	☐ Dual use research of concern
☒	☐ Plants

Methods

n/a	Involved in the study
☒	☐ ChIP-seq
☐	☒ Flow cytometry
☒	☐ MRI-based neuroimaging

Antibodies

Antibodies used

Western Blot:
Mouse anti-HA (1:1,000) (Cell Signaling, Catalog No: 2367S, Clone 6E2)
Goat anti-mouse IgG H&L HRP-conjugated secondary (1:5,000) (Abcam, Catalog No: ab6789, Polyclonal)

Immunofluorescence assays:
Rabbit anti-binding immunoglobin protein (BiP) (1:200) (kindly provided by Dr. Min Zhang)
Mouse anti-PfK13 (1:100) (Generated by Dr. Ilya Trakht in citation 52, Clone E3)
Rabbit anti-Rab11A, rat anti-Rab5B or -Rab7 (1:50) (kindly provided by Dr. Gordon Langsley)
Rabbit anti-PfACP (1:200) (kindly provided by Dr. Geoff McFadden)
Rabbit anti-PfEXP2 (1:200) (MR4)
Mouse anti-PfCRT (1:200) (Generated by Dr. Ilya Trakht in citation 92, Clone 2)
Rabbit anti-plasmepsin 2 (PM2) (1:100) (BEI Resources, Catalog No: MRA-66)
Alexa Fluor Plus 594-conjugated goat anti-rabbit IgG (H+L) secondary (1:3000) (Invitrogen, Catalog No: A32740, Polyclonal)
Alexa Fluor 594-conjugated goat anti-mouse secondary (1:2000) (Invitrogen, Catalog No: A-11005, Polyclonal)
Alexa Fluor 594-conjugated goat anti-rat secondary (1:2000) (Invitrogen, Catalog No: A-11007, Polyclonal)
Rat anti-HA (1:2000) (Millipore Sigma, Catalog No: 11867423001, Clone 3F10)
Alexa Fluor 488-conjugated goat anti-rat secondary (1:2000) (Invitrogen, Catalog No: A-11006, Polyclonal)
Mouse anti-HA (1:100) (Cell Signaling, Catalog No: 2367S, Clone 6E2)
Alexa Fluor 488-conjugated goat anti-mouse secondary (1:1000) (Invitrogen, Catalog No: A-21121, Polyclonal)

Ultrastructure Expansion Microscopy:
Rabbit anti-binding immunoglobin protein (BiP) (1:500) (kindly provided by Dr. Jeffrey Dvorin)
Mouse anti-PfK13 (1:250) (Generated by Dr. Ilya Trakht in citation 52, Clone F10)
Rat anti-Rab5B (1:250) (kindly provided by Dr. Gordon Langsley)
Rabbit anti-PfEXP2 (1:500) (MR4)
Rabbit anti-plasmepsin 2 (PM2) (1:500) (BEI Resources, Catalog No: MRA-66)
Alexa Fluor Plus 594-conjugated goat anti-rabbit IgG (H+L) secondary (1:500) (Invitrogen, Catalog No: A32740, Polyclonal)
Alexa Fluor 594-conjugated goat anti-mouse highly cross-adsorbed secondary (1:500) (Invitrogen, Catalog No: A-11032, Polyclonal)
Alexa Fluor 594-conjugated goat anti-rat secondary (1:500) (Invitrogen, Catalog No: A-11007, Polyclonal)
Rat anti-HA (1:50) (Millipore Sigma, Catalog No: 11867423001, Clone 3F10)
Alexa Fluor 488-conjugated goat anti-rat secondary (1:500) (Invitrogen, Catalog No: A-11006, Polyclonal)
Mouse anti-HA (1:250) (Cell Signaling, Catalog No: 2367S, Clone 6E2)
Alexa Fluor 488-conjugated goat anti-mouse secondary (1:500) (Invitrogen, Catalog No: A-21121, Polyclonal)

Validation

Antibodies were validated by the commercial entities or by the principal investigators that provided these reagents. The K13 antibody was from citation 52.

Eukaryotic cell lines

Policy information about cell lines and Sex and Gender in Research

Cell line source(s)

No cell lines were used that were derived from human or vertebrate models.

Authentication	We verified the identity of Plasmodium falciparum strains using Illumina whole-genome sequencing as well as SNP- and microsatellite fragment analysis-based PCR genotyping.
Mycoplasma contamination	The cell lines were not tested for Mycoplasma contamination.
Commonly misidentified lines (See ICLAC register)	No commonly misidentified lines were used in this study.

Animals and other research organisms

Policy information about studies involving animals; ARRIVE guidelines recommended for reporting animal research, and Sex and Gender in Research

Laboratory animals	Four 6-7-month-old FRG NOD human liver-chimeric (huHep) mice were purchased and shipped from the Yecuris Corporation. The mice were housed at 14.5h light/9.5h dark cycle, room temp: 68-76F, and relative humidity: 30-70%.
Wild animals	No animals were caught from the field.
Reporting on sex	We used female mice since we had small sample sizes due to the cost of the mice (n=2 per group). There are no known sex differences in liver stage parasite development, in humans and mice.
Field-collected samples	No animals were caught from the field.
Ethics oversight	All animal experiments were performed in accordance with the Animal Care and Use Committee (ACUC) guidelines and approved by the Johns Hopkins ACUC (Protocol M017H325), with modifications to a protocol reported previously (citation 74).

Note that full information on the approval of the study protocol must also be provided in the manuscript.

Plants

Seed stocks	Not applicable.
Novel plant genotypes	*Describe the methods by which all novel plant genotypes were produced. This includes those generated by transgenic approaches, gene editing, chemical/radiation-based mutagenesis and hybridization. For transgenic lines, describe the transformation method, the number of independent lines analyzed and the generation upon which experiments were performed. For gene-edited lines, describe the editor used, the endogenous sequence targeted for editing, the targeting guide RNA sequence (if applicable) and how the editor was applied.*
Authentication	*Describe any authentication procedures for each seed stock used or novel genotype generated. Describe any experiments used to assess the effect of a mutation and, where applicable, how potential secondary effects (e.g. second site T-DNA insertions, mosiacism, off-target gene editing) were examined.*

Flow Cytometry

Plots

Confirm that:

☒ The axis labels state the marker and fluorochrome used (e.g. CD4-FITC).

☒ The axis scales are clearly visible. Include numbers along axes only for bottom left plot of group (a 'group' is an analysis of identical markers).

☒ All plots are contour plots with outliers or pseudocolor plots.

☒ A numerical value for number of cells or percentage (with statistics) is provided.

Methodology

Sample preparation	Plasmodium falciparum parasite survival was assessed on an iQue PLUS, BD Accuri, or BD FACSCelesta flow cytometer using SYBR Green I and MitoTracker Deep Red FM (ThermoFisher Scientific) as stains for nuclear DNA and mitochondrial activity, respectively.
Instrument	iQue PLUS Flow Cytometer (Sartorius), BD Accuri (BD Biosciences), and BD FACSCelesta cytometer (BD Biosciences)
Software	FlowJo Version 10.10.0 (FlowJo, LLC)
Cell population abundance	Flow cytometry was used to quantify the percentage of live, fluorescently-labeled parasites in each sample. Parasitemias ranged from 0-10% at varying drug inhibitor concentrations. This method was not used for cell sorting.
Gating strategy	This is a well-established gating method routinely used to measure parasitemias, as previously described (Straimer et al. Science, 2015). Cells were first gated for red blood cells using FSC and SSC. Live parasites were determined as positive events

for BL1-A or FITC-A (SYBR Green) and RL1-A or APC-A (MitoTracker Deep Red) for the iQue or the BD FACSCelesta and BD Accuri, respectively. This corresponds to the upper right quadrant of the flow plot. Percentage of parasites was calculated as the number of live parasites events divided by the total red blood cell events for each sample. Supplementary Figure 9 shows an example of the gating strategy to quantify live parasites.

☒ Tick this box to confirm that a figure exemplifying the gating strategy is provided in the Supplementary Information.
